# In Vitro Anti-Inflammatory Activity of Methyl Derivatives of Flavanone

**DOI:** 10.3390/molecules28237837

**Published:** 2023-11-29

**Authors:** Małgorzata Kłósek, Agnieszka Krawczyk-Łebek, Edyta Kostrzewa-Susłow, Ewelina Szliszka, Joanna Bronikowska, Dagmara Jaworska, Grażyna Pietsz, Zenon P. Czuba

**Affiliations:** 1 Department of Microbiology and Immunology, Faculty of Medical Sciences, Medical University of Silesia in Katowice, Jordana 19, 41-808 Zabrze, Poland; eszliszka@sum.edu.pl (E.S.); jbronikowska@sum.edu.pl (J.B.); djaworska@sum.edu.pl (D.J.); gpietsz@sum.edu.pl (G.P.); zczuba@sum.edu.pl (Z.P.C.); 2 Department of Food Chemistry and Biocatalysis, Wrocław University of Environmental and Life Sciences, Norwida 25, 50-375 Wrocław, Poland; agnieszka.krawczyk-lebek@upwr.edu.pl (A.K.-Ł.); edyta.kostrzewa-suslow@upwr.edu.pl (E.K.-S.)

**Keywords:** cytokines, flavanones, inflammation, nitric oxide

## Abstract

Inflammation plays an important role in the immune defense against injury and infection agents. However, the inflammatory chronic process may lead to neurodegenerative diseases, atherosclerosis, inflammatory bowel diseases, or cancer. Flavanones present in citrus fruits exhibit biological activities, including anti-oxidative and anti-inflammatory properties. The beneficial effects of flavanones have been found based on in vitro cell cultures and animal studies. A suitable in vitro model for studying the inflammatory process are macrophages (RAW264.7 cell line) because, after stimulation using lipopolysaccharide (LPS), they release inflammatory cytokines involved in the immune response. We determined the nitrite concentration in the macrophage cell culture and detected ROS using chemiluminescence. Additionally, we measured the production of selected cytokines using the Bio-Plex Magnetic Luminex Assay and the Bio-PlexTM 200 System. For the first time, we have shown that methyl derivatives of flavanone inhibit NO and chemiluminescence generated via LPS-stimulated macrophages. Moreover, the tested compounds at 1–20 µM dose-dependently modulate proinflammatory cytokine production (IL-1β, IL-6, IL-12p40, IL-12p70, and TNF-α) in stimulated RAW264.7 cells. The 2′-methylflavanone (**5B**) and the 3′-methylflavanone (**6B**) possess the strongest anti-inflammatory activity among all the tested flavanone derivatives. These compounds reduce the concentration of IL-6, IL-12p40, and IL12p70 compared to the core flavanone structure. Moreover, 2′-methylflavanone reduces TNF-α, and 3′-methylflavanone reduces IL-1β secreted by RAW264.7 cells.

## 1. Introduction

Flavonoids are secondary metabolites of plants and are included in the polyphenol group as derivatives of 2-phenyl-benzo-γ-pyrone. Chemically, they are built from a fifteen-carbon structure in the C6-C3-C6 arrangement, and their carbon rings can undergo numerous modifications, including hydroxylation, methylation, or glycosylation. The word flavonoids comes from the Latin word “flavus”, meaning yellow. They accumulate in leaves, giving them their color and often their taste and smell. Flavonoids have been divided into subclasses such as chalcones, flavonols, flavan-3-ols, isoflavones, flavanones, flavones, or anthocyanins [1]. The first mentioned, i.e., chalcones, consist of two aromatic rings joined with a three-carbon, α, β-unsaturated carbonyl system. In the biosynthesis pathway of flavonoids in plants, the biosynthesis of the flavan system and other flavonoid groups begins with chalcone [2]. One of the best-known members of the chalcone class is phloretin and its glycoside phloridzin from the Malus plants, exhibiting antihyperglycemic, antioxidant, and anti-inflammatory effects [3]. Most flavonoids in the diet, besides flavan-3-ols, occur as glycosides (bound to sugar molecules) [4]. Numerous in vitro experiments, animal models, and human epidemiological trials demonstrate the high biological activity of flavonoids, including anti-cancer, cardio-protective, anti-inflammatory, immunomodulatory, or antimicrobial [5,6,7,8,9].

Flavanones (2-arylchroman-4-ones) are widely common in citrus fruits: orange, mandarin, clementine, lemon, lime, or grapefruit [10,11]. In nature, flavanones occur in the form of aglycones and glycosides, of which about 350 and 100 have been identified, respectively [12,13]. Flavones are represented by numerous naturally occurring compounds. Hesperidin (3′,5,9-dihydroxy-4′-methoxy-7-*O*-rutinosyl flavanone) and its aglycone hesperetin (5,7,3′-trihydroxy-4′-methoxy flavanone) are mainly found in mandarins, oranges, limes, and lemons. Naringin (5,7,4′-trihydroxyflavanone-7-*O*-neohesperidoside) and naringenin (5,7,4′-trihydroxyflavanone) are mainly isolated from grapefruits, whereas eriodictyol (5,7,3′,4′-tetrahydroxyflavanone) is isolated from lemons [14,15]. Other flavanone glycosides, such as eriocitrin, didymin, or narirutin, are found in citrus fruits in smaller amounts. After the consumption of the flavanone glycoside–naringin, this compound goes into the colon and is hydrolyzed into aglycones to form naringenin with the microbiota [16].

Inflammation plays an important role in the host’s immune defense against injury and infectious agents. Chronic inflammation contributes to the pathophysiology of many chronic diseases, such as atherosclerosis, diabetes, inflammatory bowel diseases, and neurodegenerative diseases, and is also related to an increased risk of cancer [17]. An active lifestyle combined with a diet rich in fruits and vegetables can help prevent pathologically prolonged inflammatory processes [18,19,20]. The bioavailability of flavonoids, their metabolism, and their biological activity depend on the substitution of functional groups and the number of hydroxyl groups. The hydroxyl group in the B ring of the flavonoid structure has the greatest impact on the ROS scavenging effect [21].

A suitable model used to study anti-inflammatory activity in vitro is RAW264.7 macrophages. Macrophages are cells of the immune system involved in the body’s defense against pathogens. They play an important role in the early inflammatory response. LPS is a potent stimulator of macrophages, under the influence of which these cells produce significant amounts of nitric oxide (NO), prostaglandin E2 (PGE2), and pro-inflammatory cytokines: IL-1β, IL-6, and TNF-α. Nitric oxide is synthesized from the amino acid L-arginine via inducible nitric oxide synthases (iNOS). NO is produced in high concentration during immunological host defense by activated macrophages, which are stimulated by LPS [22]. Therefore, inhibiting the production of inflammatory mediators, including the reduction in nitric oxide, is a very important therapeutic goal.

In recent years, flavonoids have been explored more and more intensively because these compounds show significant health-promoting effects. Several studies have shown that naturally occurring flavanones exhibit promising biological and pharmacological activities [23,24]. Flavonoide core modifications are the basis of many synthetic and structural studies to improve their biological activity. The aim of this work was to evaluate the in vitro anti-inflammatory activity of methylflavanones, including 2′-methylflavanone (**5B**), 3′-methylflavanone (**6B**), 4′-methylflavanone (**7B**), and 6-methylflavanone (**8B**), on lipopolysaccharide-induced RAW264.7 macrophages. To demonstrate this, we determined nitrite production via methyl derivatives of flavanones in LPS-stimulated RAW264.7 cells. We then detected reactive oxygen species via chemiluminescence in RAW264.7 macrophages stimulated by phorbol 12-myristate 13-acetate. In the next step, we measured the production of pro-inflammatory cytokines released by stimulated RAW264.7 cells after incubation with tested compounds. The compounds used in this study were characterized previously [25,26,27,28].

## 2. Results

### 2.1. The Cytotoxic Effect of 2′-methylflavanone (**5B**), 3′-methylflavanone (**6B**), 4′-methylflavanone (**7B**), and 6-methylflavanone (**8B**) on Activated RAW264.7 Macrophages

**Figure 1:**
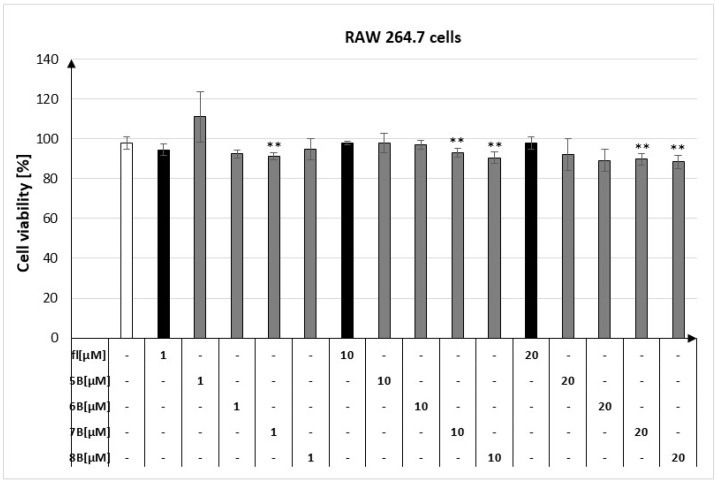
The viability of cell line RAW264.7 in the presence of flavanone (fl), 2′-methylflavanone (**5B**), 3′-methylflavanone (**6B**), 4′-methylflavanone (**7B**), and 6-methylflavanone (**8B**); ** *p* < 0.01 compared to control cells.

The RAW264.7 cells were incubated with 1–20 μM of the compounds: 2′-methylflavanone (**5B**), 3′-methylflavanone (**6B**), 4′-methylflavanone (**7B**), and 6-methylflavanone (**8B**) for 24 h. Cytotoxic effects were observed for compound **7B** at all concentrations used. (91.32 ± 1.82%–89.71 ± 2.74% of cell death). Compound **8B** was shown to be cytotoxic at the 10 μM and 20 μM used, causing 90.50 ± 2.85% of cell death and 88.46 ± 3.41% of cell death, accordingly. The other compounds used showed no cytotoxic effect on macrophages. The results of the MTT test presented as a percentage of viable cells are shown in Figure 1.

### 2.2. The Effect of the Flavanone, 2′-methylflavanone (**5B**), 3′-methylflavanone (**6B**), 4′-methylflavanone (**7B**), and 6-methylflavanone (**8B**) on Nitric Oxide (NO) Production in LPS-Stimulated RAW264.7 Macrophages

**Figure 2:**
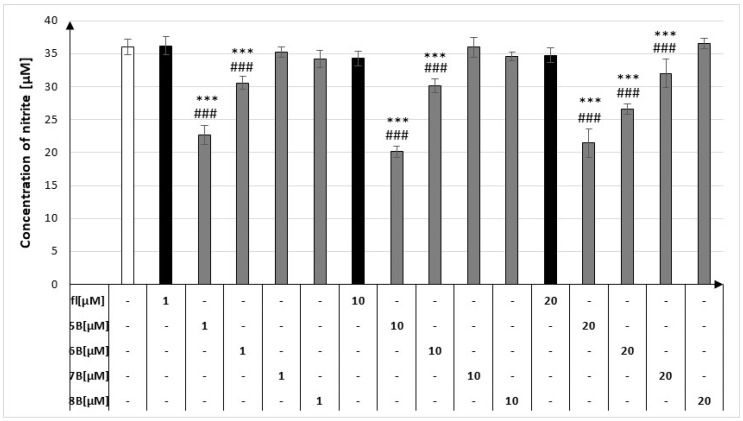
Effect of methyl derivatives of flavanones on nitrite production in LPS-stimulated RAW264.7 cells. Macrophages were incubated with 1–20 µM of tested compounds and/or LPS for 24 h. The Griess reaction assay was used to measure nitrite production. The values represent mean ± SD of three independent experiments (𝑛 = 12), *** *p* < 0.001 compared to LPS-stimulated cells; ### *p* < 0.001 compared to flavanone with LPS.

The production of NO was determined by measuring the accumulation of nitrite in the culture supernatants using the Griess reagent. In our experiment, pure flavanone (without substituents) was used as a standard. The flavanone did not reduce nitric oxide concentration, and we used it as a reference for the other flavanone derivatives. Among all the tested compounds, only **5B** and **6B** showed inhibitory activity on NO production with RAW264.7 cells incubated with LPS (Figure 2).

### 2.3. The Effect of the Flavanone, 2′-methylflavanone (**5B**), 3′-methylflavanone (**6B**), 4′-methylflavanone (**7B**), and 6-methylflavanone (**8B**) on the Chemiluminescence of Activated RAW264.7 Macrophages

**Figure 3:**
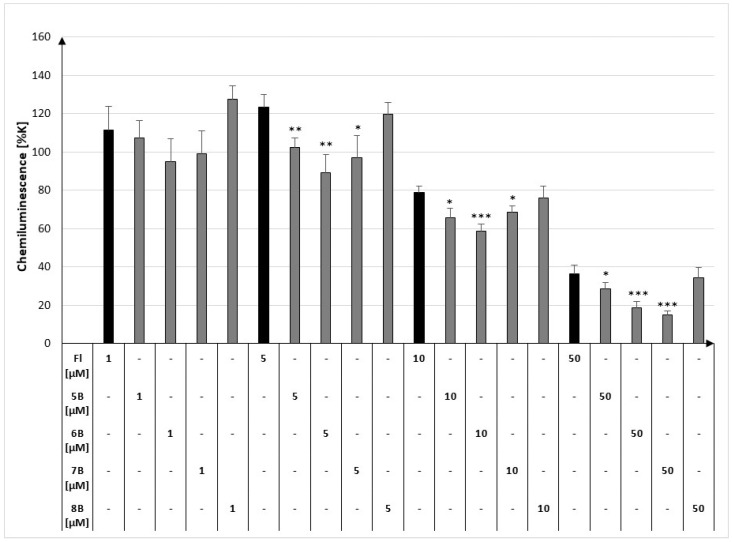
Effect of flavanone, 2′-methylflavanone (**5B**), 3′-methylflavanone (**6B**), 4′-methylflavanone (**7B**), and 6-methylflavanone (**8B**) on chemiluminescence of activated RAW264.7 macrophages; * *p* < 0.05, ** *p* < 0.01, and *** *p* < 0.001 compared to flavanone with LPS.

The study results indicated that compounds **5B** and **6B** significantly inhibited chemiluminescence in RAW264.7 macrophages stimulated via PMA. Additionally, compound **7B** showed inhibition at the highest concentration used. However, compound **8B** did not inhibit any inhibitory effect on chemiluminescence at any of the concentrations tested (Figure 3).

### 2.4. The Effect of the Flavanone, 2′-methylflavanone (**5B**), 3′-methylflavanone (**6B**), 4′-methylflavanone (**7B**), and 6-methylflavanone (**8B**) on the Release of IL-1β via Macrophages

**Figure 4:**
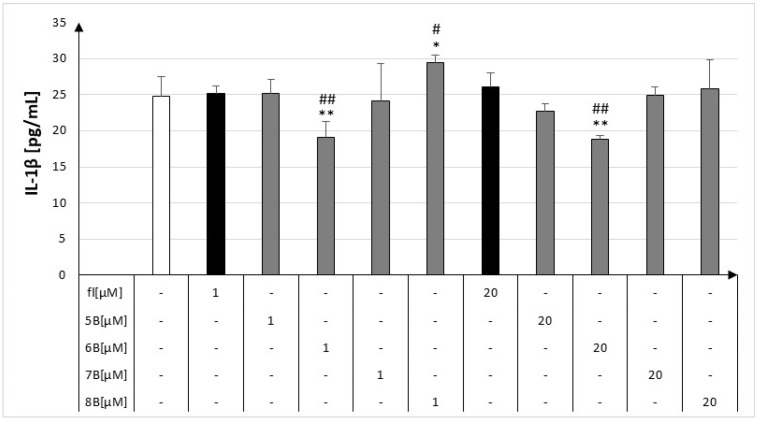
Concentrations of IL-1β in culture supernatants of cell lines RAW264.7 in the presence of flavanone (fl), 2′-methylflavanone (**5B**), 3′-methylflavanone (**6B**), 4′-methylflavanone (**7B**), and 6-methylflavanone (**8B**); # *p* < 0.05 and ## *p* < 0.01 compared to flavanone; * *p* < 0.05 and ** *p* < 0.01 compared to control (control cells and compounds incubation with LPS).

Among the tested methyl derivatives of flavanones, only compound **6B** (3′-methylflavanone) showed inhibition of the IL-1β concentration produced by RAW264.7 cells. At the 1 μM concentration, it was 19.07 ± 2.16 pg/mL, and at the 20 μM concentration, it was 18.77 ± 0.54 pg/mL of IL-1β (Figure 4). The results of Fisher’s LSD test for each cytokine are presented in Table 1 and Table 2.

### 2.5. The Effect of the Flavanone, 2′-methylflavanone, 3′-methylflavanone, 4′-methylflavanone, and 6-methylflavanone on the Release of IL-6 via Macrophages

**Figure 5:**
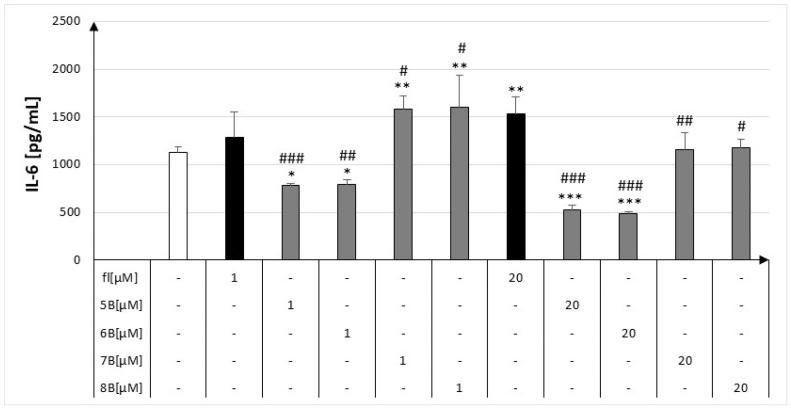
Concentrations of IL-6 in culture supernatants of macrophages in the presence of flavanone (fl), 2′-methylflavanone (**5B**), 3′-methylflavanone (**6B**), 4′-methylflavanone (**7B**), and 6-methylflavanone (**8B**); control are cells treated with LPS; # *p* < 0.05, ## *p* < 0.01, ### *p* < 0.001 compared to flavanone; * *p* < 0.05, ** *p* < 0.01 and *** *p* < 0.001 compared to control (control cells and compounds incubation with LPS).

At the highest concentration used, compounds **5B** (2′-methylflavanone) and **6B** (3′-methylflavanone) significantly inhibited the production of IL-6 by RAW264.7 cells. It was 522.70 ± 54.39 pg/mL for compound **5B** and 484.61 ± 24.18 pg/mL for compound **6B** (Figure 5).

### 2.6. The Effect of the Flavanone, 2′-methylflavanone, 3′-methylflavanone, 4′-methylflavanone, and 6-metyloflavanone on the Release of IL-12p40 Macrophages

**Figure 6:**
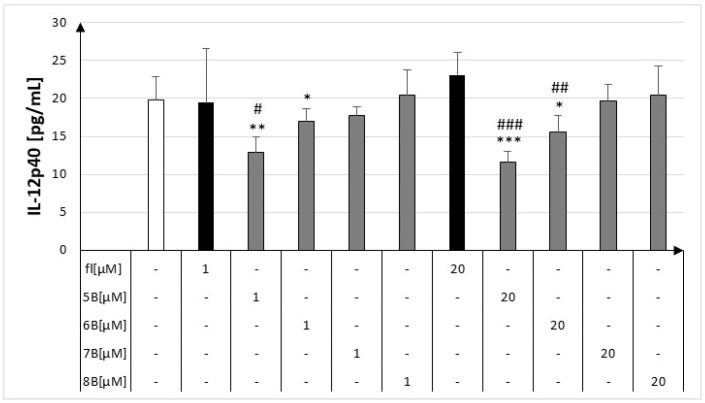
Concentrations of IL-12p40 in culture supernatants of macrophages in the presence of flavanone (fl), 2′-methylflavanone (**5B**), 3′-methylflavanone (**6B**), 4′-methylflavanone (**7B**), and 6-methylflavanone (**8B**); control are cells treated with LPS; # *p* < 0.05, ## *p* < 0.01, ### *p* < 0.001 compared to flavanone; * *p* < 0.05, ** *p* < 0.01 and *** *p* < 0.001 compared to control (control cells and compound incubations with LPS).

In our experiment, the production of IL-12p40 by RAW264.7 cells significantly decreased in the presence of compounds **5B** and **6B** at the highest concentrations used. The concentration of IL-12p40 was 11.56 ± 1.42 pg/mL for compound 5B and 15.52 ± 2.22 pg/mL for compound **6B** (Figure 6).

### 2.7. The Effect of the Flavanone, 2′-methylflavanone (**5B**), 3′-methylflavanone (**6B**), 4′-methylflavanone (**7B**), and 6-methylflavanone (**8B**) on the Release of IL-12p70 Macrophages

**Figure 7:**
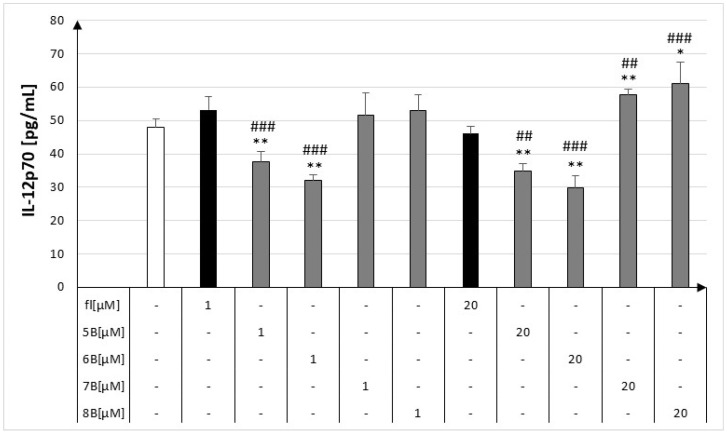
Concentrations of IL-12p70 in culture supernatants of macrophages in the presence of flavanone (fl), 2′-methylflavanone (**5B**), 3′-methylflavanone (**6B**), 4′-methylflavanone (**7B**), and 6-methylflavanone (**8B**); controls are cells treated with LPS; ## *p* < 0.01 and ### *p* < 0.001 compared to flavanone; * *p* < 0.05 and ** *p* < 0.01 compared to control (control cells and compound incubations with LPS).

Both compounds **5B** and **6B**, at both concentrations used, significantly decreased the concentration of IL-12p70 released via macrophages. For 1 μM of 2′-methylflavanone, it was 37.67 ± 2.97 pg/mL, and for 20 μM of 2′-methylflavanone, it was 34.72 ± 2.38 pg/mL. However, for 1 μM of 3′-methylflavanone, it was 31.96 ± 1.80 pg/mL, and for 20 μM of 3′-methylflavanone, it was 29.81 ± 3.67 pg/mL (Figure 7).

### 2.8. The Effect of the Flavanone, 2′-methylflavanone, 3′-methylflavanone, 4′-methylflavanone, and 6-methylflavanone on the Release of TNF-α Macrophages

**Figure 8:**
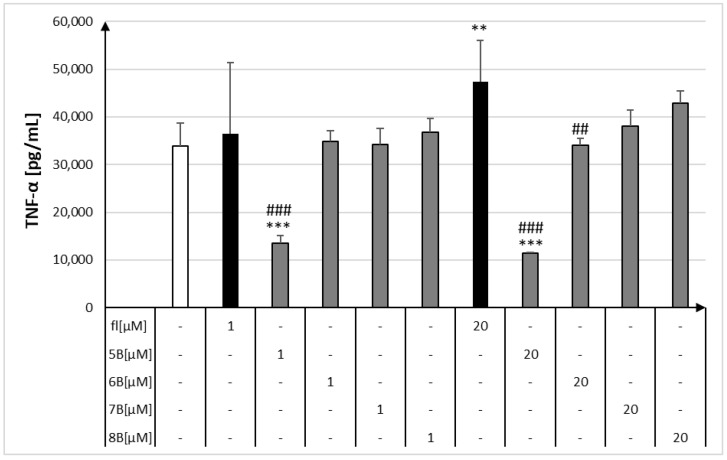
Concentrations of TNF-α in culture supernatants of macrophages in the presence of flavanone (fl), 2′-methylflavanone (**5B**), 3′-methylflavanone (**6B**), 4′-methylflavanone (**7B**), and 6-methylflavanone (**8B**); control are cells treated with LPS; ## *p* < 0.01 and ### *p* < 0.001 compared to flavanone; ** *p* < 0.01 and *** *p* < 0.001 compared to control (control cells and compound incubations with LPS).

At a concentration of 20 μM, compound **5B** (2′-methylflavanone) significantly decreased the concentration of TNF-α to 11,405.34 ± 150.03 pg/mL. However, the other tested compounds did not significantly affect TNF-α activity (Figure 8).

## 3. Discussion

Inflammation is an important risk factor in the pathogenesis of chronic diseases. Cells of the immune system, including lymphocytes, dendritic cells, and macrophages, play a role in both physiological and pathological processes within the body, including inflammatory reactions. Macrophages exhibit high sensitivity to inflammation triggers, such as bacterial endotoxin-lipopolysaccharide (LPS). In response to LPS in the immune system, they release reactive oxygen species (ROS), reactive nitrogen species (RNS), nitric oxide (NO), and pro-inflammatory cytokines. The excessive production of ROS is termed ‘oxidative stress’, which leads to lipid peroxidation and damage to DNA, RNA, and proteins [29]. In vitro and in vivo studies have demonstrated that flavonoids possess anti-inflammatory properties by inhibiting nitric oxide synthase in macrophages. Flavonoids also inhibit cyclooxygenase, lipoxygenase, or xanthine oxidase, which generate ROS [30].

The compounds under the subject of our research are methyl derivatives of flavanones. All compounds used in the study were obtained via a two-step chemical synthesis. The first step involved the Claisen–Schmidt condensation reaction, resulting in chalcones (**5A**, **6A**, **7A**, and **8A**). The second step was the cyclization of these compounds in the presence of sodium acetate, resulting in flavanones **5B**, **6B**, **7B**, and **8B** [25,26,27,28]. We determined the activity of methyl derivatives of flavanone by measuring their cytotoxicity on macrophage cell lines, the concentration of nitrite released via macrophages after stimulation of tested compounds, the determined chemiluminescence, and the released proinflammatory cytokines.

In our experiment, we have shown that only 2′-methylflavanone (**5B**) and 3′-methylflavanone (**6B**) caused a decrease in NO production via LPS-stimulated macrophages across all examined concentrations. The mechanism of action of these compounds may depend on the position of the methyl groups in the B ring. During stimulation, phagocytic cells release significant amounts of reactive oxygen species, a reaction that can be measured in vitro through chemiluminescence. This process of releasing reactive oxygen species is accompanied with the emission of photons.

In our experiment, we illustrated that compounds **5B** and **6B** mainly inhibited chemiluminescence in RAW264.7 cells stimulated via PMA. Flavanones are generally weaker free radical scavengers compared to flavonols. Based on the results of the viability test, we selected low concentrations of the tested compounds. We stimulated macrophages with LPS and/or tested methyl derivatives of flavanone to determine the production of cytokine concentrations via activated macrophages. In this study, we, for the first time, determined the impact of methyl derivatives of flavanones on the production of IL-1β, IL-6, IL-12p40, IL-12p70, and TNF-α. The cytokines selected by us are attributed to playing an important role in the pathogenesis of some inflammatory diseases. Interleukin 1β, along with TNF-α (tumor necrosis factor α) and interleukin-6, is considered a major pro-inflammatory cytokine. IL-1β is induced during the acute inflammatory process, promoting the up-regulation of adhesion receptors on endothelial and immune cells, which triggers leukocyte infiltration into infection sites [31]. IL-6 has similar effects to IL-1β and TNF-α, but unlike them, it has a longer half-life, reaching its highest serum concentration 2–3 h after stimulation by endotoxin.

Interleukin 12p70 (IL-12) is a heterodimeric cytokine consisting of two monomeric subunits, p40 and p35. It is mainly produced by monocytes and macrophages and plays a role in the regulation of the differentiation of naïve T cells into Th1 cells. IL-12p70 is linked to the pathogenesis of chronic inflammatory diseases, such as Crohn’s disease or rheumatoid arthritis [32,33]. Ichikawa D. et al. investigated the effect of catechins on IL-12p40 production in murine macrophages induced by bacterial lipopolysaccharide. They demonstrated that among the tested catechins, the most potent inhibitor was epigallocatechin gallate (EGCG), which led to a decrease in IL-12p40 production [34]. Moreover, it inhibited LPS-induced degradation of IκBα along with concomitant inhibition of nuclear protein binding to the NF-κB site and synthesis of IRF-1. Our study showed that the compounds **5B** and **6B**, at a concentration of 20 µM, inhibited the production of IL-12p40 in RAW264.7 cells.

The mechanisms of action of flavanones present in citrus fruits have been elucidated through in vitro cell cultures and animal studies, documenting their anti-inflammatory effects. Sakata K. et al. investigated the anti-inflammatory activity of hesperidin. They treated RAW 264.7 cells with 0.2 μg/mL LPS and 10, 20, and 30 μM of hesperidin [35]. The inhibition of NO production by macrophages was observed in a dose-dependent manner. Additionally, treatment of cells with hesperidin suppressed the production of PGE2 and the expression of the iNOS protein. Another flavanone, neohesperidin (hesperetin-7-neohesperidoside), has demonstrated anti-inflammatory effects by reducing the levels of proinflammatory cytokines IL-1β, IL-6, IL-8, TNF-α, and metalloproteinases MMP-3, MMP-9, and MMP-13 in TNF-α-stimulated human rheumatoid arthritis fibroblast-like synoviocytes. The authors suggested that neohesperidin may hold therapeutic potential in rheumatoid arthritis [36]. Neohesperidin serves as a natural source for the synthesis of neohesperidin dihydrochalcone (NHDC), a low-calorie synthetic sweetener. In vitro and animal model studies were conducted to evaluate the possible mutagenic effects and safety of dietary administration of this sweetener. The data obtained from this study did not reveal any carcinogenic or teratogenic effects that could be linked to NHDC [37,38].

Naringenin effectively inhibits the production of pro-inflammatory cytokines by activated macrophages and reduces the production of nitrate and nitrite [39]. In both in vivo animal models and in vitro studies, naringenin has been demonstrated to downregulate the expression of IL-1β, IL-6, TNF-α, iNOS, and COX-2 through the attenuation of the NF-κB pathway and the activation of the AMPK (AMP-activated protein kinase) [39]. Kanno S. et al. have shown that a different flavanone, naringin, suppresses NO production in LPS-stimulated macrophages (RAW 264.7). Moreover, naringin suppresses the expression of inflammatory gene products, such as iNOS, COX-2, TNF-α, and IL-6, along with the transcriptional activity of NF-κB [40]. In animal models of inflammation, naringin modulates the expression of TNF-α, iNOS, and Nrf2 (nuclear factor erythroid 2-related factor 2) [41]. The extract of Citrus wilsonii Tanaka, containing high amounts of naringin, significantly inhibits the production of prostaglandin E2 (PGE2) and secretion of cyclooxygenase-2 (COX-2) and suppresses the mRNA expression of inflammatory mediators such as COX-2, IL-1β, IL-6, and TNF-α in LPS-induced RAW 264.7 macrophages [42].

Another flavanone with established anti-inflammatory properties is pinocembrin (5, 7-dihydroxyflavanone), one of the most abundant flavonoids in propolis [43]. Soromou L. et al. assessed its anti-inflammatory effects in LPS-stimulated RAW 264.7 mouse macrophage cells and in BALB/c mice, which are LPS-induced acute lung injury [44]. Pinocembrin significantly decreased the production of IL-1β, IL-6, and TNF-α while significantly increasing IL-10 levels. Additionally, this flavanon inhibited the phosphorylation of IκBα, ERK1/2, JNK, and p38MAP activation. Kim et al. investigated the anti-inflammatory activity of sakuranetin (4,5-dihydroxy-7-methoxyflavanone), an O-methylated derivative of naringenin [45]. They stimulated mouse peritoneal macrophages with IFN-𝛾 and LPS using sakuranetin at concentrations ranging from 10 μM to 100 μM and observed a dose-dependent reduction in NO release via sakuranetin. Furthermore, sakuranetin suppressed the synthesis of iNOS and COX2 in LPS/IFN-𝛾-stimulated macrophages and inhibited the secretion of TNF-α, IL-6, and IL-12 in these examined cells stimulated via LPS.

Citrus fruits contain a high amount of 2′-hydroxyflavanone, a well-documented bioactive compound [46]. H. Sonowal demonstrated that 2′-hydroxyflavanone prevents the production of reactive oxygen species and nitric oxide via LPS-induced macrophages [47]. It also prevents lipid peroxidation and the loss of mitochondrial membrane potential in RAW264.7 cells. This compound inhibits the expression of IL-2, IL-10, IL-12p40, TNF-α, LIX, IL-15, IL-17, MCP-1, and eotaxin induced via LPS in macrophages. Additionally, it prevents LPS-phosphorylation, nuclear translocation, and DNA-binding of NF-κB.

For the first time, we have shown that methyl derivatives of flavanone inhibit nitric oxide and chemiluminescence generated via LPS-stimulated macrophages. Our study demonstrated that 2′-methylflavanone (**5B**) and 3′-methylflavanone (**6B**) possess the strongest anti-inflammatory activity among all the tested flavanone derivatives. These compounds reduce the concentration of IL-6, IL-12p40, and IL12p70 compared to the core flavanone structure. Moreover, 2′-methylflavanone reduces TNF-α, while 3′-methylflavanone reduces IL-1β secreted by RAW264.7 cells.

## 4. Materials and Methods

### 4.1. General Procedure for the Synthesis of Methylflavanones

**Scheme 1:**
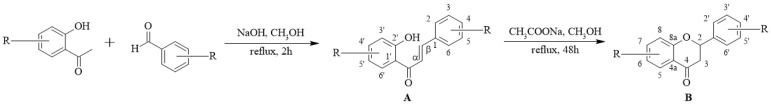
Synthesis of flavonoid compounds (chalcones **5A**–**8A** and flavanones **5B**–**8B**).

The compounds for the experiments were obtained via a two-step synthesis (Scheme 1). In the first step, chalcones (**5A**, **6A**, **7A**, and **8A**) were synthesized with the Claisen–Schmidt condensation reaction from commercially available 2-hydroxyacetophenone (or 2-hydroxy-5-methylacetophenone in the case of compound **8B**) and benzaldehyde (in the case of compound **8B**) or 2-methylbenzaldehyde (in the case of compound **5B**) or 3-methylbenzaldehyde (in the case of compound **6B**) or 7-methylbenzaldehyde (in the case of compound **7B**) according to the method previously described. The chemical syntheses of chalcones and their spectroscopic characteristics (NMR and MS) were described in the previous papers: [25] (chalcone **5A**), [26] (chalcone **7A**), and [27] (chalcone **8A**). The NMR and MS data of chalcone **6A** are shown in the Supplementary Materials (Figures S1–S12). Four flavanones (**5B**, **6B**, **7B**, and **8B**) were obtained via cyclization of the corresponding 2′-hydroxychalcones (**5A**, **6A**, **7A**, and **8A**) in the presence of sodium acetate according to the method previously described. Their NMR and MS data were described in the previous papers: [28] (flavanone **5B**), [26] (flavanone **7B**), and [25] (flavanone **8B**). The NMR and MS data of flavanone **6B** are shown in the Supplementary Materials (Figures S13–S23). The structures of the obtained flavonoids are shown below in Table 3.

The chosen flavanones with a methyl group were analyzed using computational simulations based on their structural formulas with the SwissADME online tool. This tool conducts in silico simulations to assess the pharmacokinetics, drug-likeness, and medicinal chemistry suitability of small molecules. The results indicated that these compounds are water-soluble, unable to penetrate the blood–brain barrier, and likely to be highly absorbed in the gastrointestinal tract. The Abbott bioavailability score (ABS), indicating a compound’s probability of having >10% bioavailability in rats or measurable Caco-2 cell permeability, was 0.55 for all tested compounds. This suggests that 55% of these flavonoids might have a bioavailability higher than 10%. Additionally, the tested compounds adhered to Lipinski’s rule, Veber’s rule, and Ghose’s filter, confirming their drug-likeness. Furthermore, these flavonoids did not exhibit any PAINS alerts, indicating their suitability for in vitro tests as potential drugs.

### 4.2. Cell Culture

In this experiment, a mouse peritoneal macrophage cell line, RAW 264.7, from ATCC (American Type Culture Collection, Manassas, VA, USA) was used. These cells were established from a tumor in a male mouse induced with the Abelson murine leukemia virus. Cell culture was carried out continuously at 37 °C in an atmosphere with 5% CO_2_ and 100% relative humidity. Macrophages were cultured using DMEM (Dulbecco’s modified eagle’s minimum essential medium) with 10% heat-inactivated fetal bovine serum (FBS), 100 IU/mL of penicillin, and 100 μg/mL of streptomycin. Adhered macrophage cells were scraped off with the scrapers to detach them from the bottom of the dish. They were then made into suspensions for further experiments. The density of the cell suspension was evaluated via microscopy using a Bürker chamber. A suspension of cells from the RAW264.7 line with a density of 1 × 10^6^ in 1 mL of medium was used for the experiments.

### 4.3. Cell Viability Assay

The potential toxicity of flavanone methyl-derivatives was evaluated using the 3-(4,5-dimethylthiazol-2-yl)-2,5 diphenyltetrazolium (MTT) assay, as described in previous studies [48]. This assay relies on viable cells cleaving the tetrazolium salt MTT (provided by Sigma Chemical Company, St. Louis, MO, USA) to produce a blue formazan dye. RAW264.7 cells (1 × 10^6^/mL) were seeded into each well of a 96-well plate 20 h before the experiments. The cells were treated for 24 h with flavanone (fl), 2′-methylflavanone (**5B**), 3′-methylflavanone (**6B**), 4′-methylflavanone (**7B**), and 6-methylflavanone (**8B**) (dissolved in DMSO) at concentrations of 1 µM, 10 μM, and 20 μM with or without LPS. After this time, the supernatants were collected, and they were frozen at −80 °C (afterward, cytokines were determined in the supernatants). After aspirating the medium, 180 µL of culture medium and 20 μL of MTT solutions (5 mg/mL) were added to each well and incubated for 4 h. The resulting formazan crystals were dissolved in 100% dimethyl sulfoxide (DMSO). All the reagents were obtained from Sigma Chemical Company (St. Louis, MO, USA). Control samples included native cells and medium alone. The spectrophotometric absorbance of each well was assessed using a microplate reader (EonTM Microplate Spectrophotometer, BioTek, Winooski, VT, USA) at a wavelength of 550 nm. The cytotoxicity, as the percentage of cell death, was calculated using the formula: percent cytotoxicity (cell death) = [1 − (absorbance of experimental wells/absorbance of control wells)] × 100%.

### 4.4. Nitric Oxide Assay

RAW 264.7 macrophages at 1 × 10^6^/mL were adhered to 96-well plates. After 4 h, 1–20 µM of methyl-derivatives of flavanones and/or 200 ng/mL of LPS were added. After 20 h, the supernatants were collected, and the nitric oxide concentration was determined. The concentration of nitric oxide released by macrophages was determined by measuring the accumulation of nitrite, the stable end product, in the culture supernatant according to the Griess reaction. To 100 μL volume of culture supernatant from each well, 100 μL of Griess reagent was added and incubated for 15 min at room temperature. Absorbance was read at 530 nm in a BioTek spectrophotometer (Eon Microplate, Winooski, VT, USA). Nitrite concentration in the medium was calculated using sodium nitrite as a standard. Nitrite was not detectable in cell-free medium [49].

### 4.5. Detection of ROS via Chemiluminescence

The RAW 264.7 cells were incubated with 1–50 μM: flavanone (fl), 2′-methylflavanone (**5B**), 3′-methylflavanone (**6B**), 4′-methylflavanone (**7B**), and 6-methylflavanone (**8B**) for 30 min. Then, a luminol (Sigma Chemical Company) solution was added to wells containing 2 × 10^5^ cells/well to obtain a 1.13 × 10^−4^ M final concentration. After 5 min, an 8 × 10^−7^ M phorbol 12-myristate 13-acetate (PMA) solution (Sigma Chemical Company) was used. The final volume of each well was 200 μL. The chemiluminescence was determined after stimulation with PMA for 30 min. The measurement was made using a LB 960 CentroXS3 microplate luminometer (Berthold Technologies GmbH, Wildbad, Germany) [49].

### 4.6. Quantification of the IL-1β, IL-6, IL-12p40, IL-12p70, and TNF-α Concentrations

The concentrations of IL-1β, IL-6, IL-12p40, IL-12p70, and TNF-α released from RAW264.7 cells were determined after 24 h of stimulation with flavanone (fl), 2′-methylflavanone (**5B**), 3′-methylflavanone (**6B**), 4′-methylflavanone (**7B**), and 6-methylflavanone (**8B**) with or without LPS in the supernatants of cell culture. The measurements were conducted with the Bio-Plex Magnetic Luminex Assay (Bio-Rad Inc., Hercules, CA, USA) and the Bio-Plex^TM^ 200 System (Bio-Rad Laboratories, Inc., Hercules, CA, USA). Color-coded in shades of red, sets of magnetic beads coated with antibodies specific for the analytes were added to the supernatants of cell cultures and standards combined with specific analytes. Bead-linked analytes were detected after the addition of a cocktail of biotinylated antibodies, which afterwards reacted with the streptavidin phycoerythrin conjugate. After each incubation period, an ELx 50 magnetic washer (BioTek, Winooski, Vermont, USA) was used to wash out the ferromagnetic beads. The quantification was performed using the BioRad System 200 instrument, whose operation is based on a flow cytometry technique that uses two lasers. The concentrations of analyte were read from the curves according to appropriate standards using the instrument control software and analysis of the results. The experiments were repeated fourfold [50,51].

### 4.7. Statistical Analysis

In the case of cytotoxicity determination, the values represent means ± SD obtained from three independent experiments in quadruplicate (*n* = 12). For chemiluminescence, the values represent means ± SD obtained from three independent experiments in triplicate (*n* = 9). The distribution of the data was verified for normality with the Shapiro–Wilk test. Significant differences were analyzed using the Student’s *T*-test, and *p*-values < 0.05 were considered significant. Results of the nitric oxide assay were obtained from three independent experiments in triplicate (*n* = 9) and cytokine determination. The values represent means ± SD obtained from three independent experiments (*n* = 3). The homogeneity of variance was tested with Levene’s test. The statistical analyses were performed using STATISTICA 13.1 software (StatSoft Inc., Tulsa, OK, USA). A one-way ANOVA was used to compare the effect of methyl derivatives of flavanones among tested compounds and controls, as well as among tested compounds and flavanones.

## 5. Conclusions

A high intake of flavonoids, including flavanones, may offer protection against oxidation, inflammation, and chronic diseases. Based on the results of our research, we can conclude that 2′-methylflavanone (**5B**) and 3′-methylflavanone (**6B**) have shown anti-inflammatory potential and may have beneficial health effects. In the future, we will continue our study to determine other inflammatory factors that can be affected by 2′-methylflavanone (**5B**), 3′-methylflavanone (**6B**), 4′-methylflavanone (**7B**), and 6-methylflavanone (**8B**). We will assess the effect of methyl derivatives of flavanone in in vivo studies.

## Figures and Tables

**Table 1:** The effect of methyl derivatives of flavanone on the production of IL-1β, IL-6, IL-12p40, IL-12p70, and TNF-α was compared to the control in LPS-stimulated RAW264.1 cells (*n* = 3). Statistical significance was analyzed using Fisher’s LSD test. Results marked in red are statistically significant in Fisher’s LSD test. Multivariate Tests of Significance (F = 7.499, *p* < 0.05).

Sample	IL-1β		IL-6		IL-12p40		IL-12p70		TNF-α	
	CV	*p*	CV	*p*	CV	*p*	CV	*p*	CV	*p*
Control	10.704364		5.478535		13.342202		5.166537		14.357457	
**5B** 1 μM	8.070487	0.880882	2.756384	0.014881	16.519107	0.001031	7.895512	0.003850	11.322122	0.000296
**5B** 20 μM	4.570054	0.318805	10.405476	0.000133	12.298108	0.000307	6.868333	0.000410	1.315403	0.000095
**6B** 1 μM	11.353243	0.009652	6.321492	0.018428	9.332954	0.038380	5.639210	0.000050	6.345997	0.826990
**6B** 20 μM	2.860606	0.006865	4.989167	0.000066	14.294118	0.010886	12.327883	0.000010	4.260733	0.974889
**7B** 1 μM	21.495385	0.757877	8.755781	0.002296	6.472807	0.065044	12.839975	0.264156	9.463668	0.927038
**7B** 20 μM	4.570054	0.963008	14.867259	0.804608	11.607130	0.232104	2.927687	0.005071	8.586474	0.373381
**8B** 1 μM	3.760656	0.032619	20.741240	0.001515	16.148254	0.362711	8.665186	0.121969	7.928790	0.531949
**8B** 20 μM	15.244568	0.612231	7.973248	0.735847	19.096473	0.365265	10.445151	0.000408	6.080636	0.069246
Flavanone 1 μM	4.485925	0.875184	21.132576	0.253626	36.649072	0.205639	7.560869	0.111074	40.729791	0.571884
Flavanone 20 μM	7.669079	0.538352	11.567501	0.005065	13.151849	0.946897	4.687945	0.570728	18.376227	0.008844

**Table 2:** The effect of methyl derivatives of flavanone on the production of IL-1β, IL-6, IL-12p40, IL-12p70, and TNF-α compared to flavanone (1 μM and 20 μM, accordingly) in LPS-stimulated RAW264.1 cells (*n* = 3). Statistical significance was analyzed using Fisher’s LSD test. Results marked in red are statistically significant in Fisher’s LSD test. Multivariate Tests of Significance (F = 7.499, *p* < 0.05).

Sample	IL-1β		IL-6		IL-12p40		IL-12p70		TNF-α	
	CV	*p*	CV	*p*	CV	*p*	CV	*p*	CV	*p*
Flavanone 1 μM	4.485925		21.132576		36.649072		7.560869		40.729791	
Control	10.704364	0.875184	5.478535	0.253626	13.342202	0.205639	5.166537	0.111074	14.357457	0.571884
**5B** 1 μM	8.070487	0.994230	2.756384	0.000947	16.519107	0.021488	7.895512	0.000069	11.322122	0.000073
**6B** 1 μM	11.353243	0.006699	6.321492	0.001197	9.332954	0.378589	5.639210	0.000001	6.345997	0.727688
**7B** 1 μM	21.495385	0.642240	8.755781	0.032999	6.472807	0.530175	12.839975	0.612198	9.463668	0.635104
**8B** 1 μM	3.760656	0.045369	20.741240	0.022795	16.148254	0.711464	8.665186	0.959332	7.928790	0.951763
Flavanone 20 μM	7.669079		11.567501		13.151849		4.687945		18.376227	
Control	10.704364	0.538352	5.478535	0.005065	13.342202	0.946897	5.166537	0.570728	14.357457	0.008844
**5B** 20 μM	4.570054	0.114164	10.405476	0.000000	12.298108	0.000260	6.868333	0.001660	1.315403	0.000000
**6B** 20 μM	2.860606	0.001562	4.989167	0.000000	14.294118	0.009340	12.327883	0.000039	4.260733	0.009512
**7B** 20 μM	4.570054	0.569024	14.867259	0.009041	11.607130	0.208327	2.927687	0.001286	8.586474	0.062300
**8B** 20 μM	15.244568	0.912729	7.973248	0.011128	19.096473	0.332055	10.445151	0.000100	6.080636	0.346267

**Table 3:** Structures of flavanones **5B**–**8B**.

Compound	Structure	R_1_	R_2_	R_3_	R_4_
**5B**	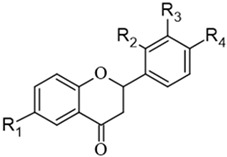	H	CH_3_	H	H
**6B**		H	H	CH_3_	H
**7B**		H	H	H	CH_3_
**8B**		CH_3_	H	H	H

## Data Availability

Data are contained within the article and Supplementary Materials.

## Supplementary Materials

The following supporting information can be downloaded at https://www.mdpi.com/article/10.3390/molecules28237837/s1, Figure S1. MS analysis of 2’-hydroxy-3-methylchalcone (**6A**); Figure S2. ^1^H NMR spectrum (*δ,* acetone-d6, 600 MHz) of 2’-hydroxy-3-methylchalcone (**6A**); Figure S3. ^1^H NMR spectrum expansion (*δ,* acetone-d6, 600 MHz) of 2’-hydroxy-3-methylchalcone (**6A**); Figure S4. ^13^C NMR spectrum (*δ*, acetone-d6, 151 MHz) of 2’-hydroxy-3-methylchalcone (**6A**); Figure S5. ^13^C NMR spectrum expansion (*δ*, acetone-d6, 151 MHz) of 2’-hydroxy-3-methylchalcone (**6A**); Figure S6. COSY contour map –^1^H x^1^H of 2’-hydroxy-3-methylchalcone (**6A**); Figure S7. COSY contour map –^1^H x^1^H expansion of 2’-hydroxy-3-methylchalcone (**6A**); Figure S8. HSQC contour map –^1^H x^13^C of 2’-hydroxy-3-methylchalcone (**6A**); Figure S9. HSQC contour map –^1^H x^13^C expansion of 2’-hydroxy-3-methylchalcone (**6A**); Figure S10. HMBC contour map –^1^H x^13^C of 2’-hydroxy-3-methylchalcone (**6A**); Figure S11. HMBC contour map –^1^H x^13^C expansion of 2’-hydroxy-3-methylchalcone (**6A**); Figure S12. HMBC contour map –^1^H x^13^C expansion of 2’-hydroxy-3-methylchalcone (**6A**); Figure S13. MS analysis of 3’-methylflavanone (**6B**); Figure S14. ^1^H NMR spectrum (*δ*, acetone-d6, 600 MHz) of 3’-methylflavanone (**6B**); Figure S15. ^1^H NMR spectrum expansion (*δ*, acetone-d6, 600 MHz) of 3’-methylflavanone (**6B**); Figure S16. ^13^C NMR spectrum (*δ*, acetone-d6, 151 MHz) of 3’-methylflavanone (**6B**); Figure S17. ^13^C NMR spectrum expansion (*δ*, acetone-d6, 151 MHz) of 3’-methylflavanone (**6B**); Figure S18. COSY contour map –^1^H x^1^H of 3’-methylflavanone (**6B**); Figure S19. COSY contour map –^1^H x^1^H expansion of 3’-methylflavanone (**6B**); Figure S20. HSQC contour map –^1^H x^13^C of 3’-methylflavanone (**6B**); Figure S21. HSQC contour map –^1^H x^13^C expansion of 3’-methylflavanone (**6B**); Figure S22. HMBC contour map –^1^H x^13^C expansion of 3’-methylflavanone (**6B**); Figure S23. HMBC contour map –^1^H x^13^C expansion of 3’-methylflavanone (**6B**); Table S1. The effect of methyl-derivatives of flavanone on the production IL-1β in compared to control in LPS stimulated RAW264.1 cells (*n* = 3). Statistical significance was analysed using Fisher’s LSD test. Results marked in red are statistically significant in Fisher’s LSD test. Multivariate Tests of Significance (F = 7.499, *p* < 0.05); Table S2. The effect of methyl-derivatives of flavanone on the production IL-6 in compared to control in LPS stimulated RAW264.1 cells (*n* = 3). Statistical significance was analysed using Fisher’s LSD test. Results marked in red are statistically significant in Fisher’s LSD test. Multivariate Tests of Significance (F = 7.499, *p* < 0.05); Table S3. The effect of methyl-derivatives of flavanone on the production IL-12p40 in compared to control in LPS stimulated RAW264.1 cells (*n* = 3). Statistical significance was analysed using Fisher’s LSD test. Results marked in red are statistically significant in Fisher’s LSD test. Multivariate Tests of Significance (F = 7.499, *p* < 0.05); Table S4. The effect of methyl-derivatives of flavanone on the production IL-12p70 in compared to control in LPS stimulated RAW264.1 cells (*n* = 3). Statistical significance was analysed using Fisher’s LSD test. Results marked in red are statistically significant in Fisher’s LSD test. Multivariate Tests of Significance (F = 7.499, *p* < 0.05); Table S5. The effect of methyl-derivatives of flavanone on the production TNF-α in compared to control in LPS stimulated RAW264.1 cells (*n* = 3). Statistical significance was analysed using Fisher’s LSD test. Results marked in red are statistically significant in Fisher’s LSD test. Multivariate Tests of Significance (F = 7.499, *p* < 0.05); Table S6. Average, standard deviations, statistical significance of cytotoxic activity of methyl-derivatives of flavanone. Statistical significance was calculated using *t*-test (Table S3); Table S7. Average, standard deviations, statistical significance of chemiluminescence of methyl-derivatives of flavanone. Statistical significance was calculated using *t*-test (Table S5); Table S8. The effect of methyl-derivatives of flavanone on the concentration of nitrite in compared to control in LPS stimulated RAW264.1 cells (*n* = 3). Statistical significance was analysed using Fisher’s LSD test. Results marked in red are statistically significant in Fisher’s LSD test. Multivariate Tests of Significance (F = 7.499, *p* < 0).
