# Supplementary material for: In Vitro Anti-Inflammatory Activity of Methyl Derivatives of Flavanone
Source: Molecules. 2023 Nov 29;28(23):7837. doi: 10.3390/molecules28237837 (PMC10708004; doi:10.3390/molecules28237837)
Supplement: Supplementary file 1 [file molecules-28-07837-s001.zip › molecules-2645549-supplementary.pdf]

molecules

MDPI

Supplementary Materials

In Vitro Anti-Inflammatory Activity of Methyl Derivatives of Flavanone

Małgorzata Kłósek [1,*], Agnieszka Krawczyk-Łebek [2], Edyta Kostrzewa-Susłow [2], Ewelina Szliszka [1], Joanna Bronikowska [1], Dagmara Jaworska [1], Grażyna Pietsz [1] and Zenon P. Czuba [1]

[1] Department of Microbiology and Immunology, Faculty of Medical Sciences, Medical University of Silesia in Katowice, Jordana 19, 41-808 Zabrze, Poland; eszliszka@sum.edu.pl (E.S.); jbronikowska@sum.edu.pl (J.B.); djaworska@sum.edu.pl (D.J.); gpietsz@sum.edu.pl (G.P.); zczuba@sum.edu.pl (Z.P.C.)

[2] Department of Food Chemistry and Biocatalysis, Wrocław University of Environmental and Life Sciences, Norwida 25, 50-375 Wrocław, Poland; agnieszka.krawczyk-lebek@upwr.edu.pl (A.K.-Ł.); edyta.kostrzewa-suslow@upwr.edu.pl (E.K.-S.)

* Correspondence: mklosek@sum.edu.pl; Tel./Fax: +48-322-722-554

Citation: Kłósek, M.; Krawczyk-Łebek, A.; Kostrzewa-Susłow, E.; Szliszka, E.; Bronikowska, J.; Jaworska, D.; Pietsz, G.; Czuba, Z.P. In Vitro Anti-Inflammatory Activity of Methyl Derivatives of Flavanone. *Molecules* **2023**, *28*, 7837. https://doi.org/10.3390/molecules28237837

Academic Editors: Athina Geronikaki and H. P. Vasantha Rupasinghe

Received: 18 September 2023
Revised: 26 November 2023
Accepted: 27 November 2023
Published: 29 November 2023

Copyright: © 2023 by the authors. Submitted for possible open access publication under the terms and conditions of the Creative Commons Attribution (CC BY) license (https://creativecommons.org/licenses/by/4.0/).

Content

Figure S1. MS analysis of 2′-hydroxy-3-methylchalcone (**6A**)

Figure S2. ^{1}H NMR spectrum (*δ*, acetone-d6, 600 MHz) of 2′-hydroxy-3-methylchalcone (**6A**)

Figure S3 ^{1}H NMR spectrum expansion (*δ*, acetone-d6, 600 MHz) of 2′-hydroxy-3-methylchalcone (**6A**)

Figure S4. ^{13}C NMR spectrum (*δ*, acetone-d6, 151 MHz) of 2′-hydroxy-3-methylchalcone (**6A**)

Figure S5. ^{13}C NMR spectrum expansion (*δ*, acetone-d6, 151 MHz) of 2′-hydroxy-3-methylchalcone (**6A**)

Figure S6. COSY contour map – ^{1}H x ^{1}H of 2′-hydroxy-3-methylchalcone (**6A**)

Figure S7. COSY contour map – ^{1}H x ^{1}H expansion of 2′-hydroxy-3-methylchalcone (**6A**)

Figure S8. HSQC contour map – ^{1}H x ^{13}C of 2′-hydroxy-3-methylchalcone (**6A**)

Figure S9. HSQC contour map – ^{1}H x ^{13}C expansion of 2′-hydroxy-3-methylchalcone (**6A**)

Figure S10. HMBC contour map – ^{1}H x ^{13}C of 2′-hydroxy-3-methylchalcone (**6A**)
Figure S11. HMBC contour map – ^{1}H x ^{13}C expansion of 2′-hydroxy-3-methylchalcone (**6A**)
Figure S12. HMBC contour map – ^{1}H x ^{13}C expansion of 2′-hydroxy-3-methylchalcone (**6A**)
Figure S13. MS analysis of 3′-methylflavanone (**6B**)
Figure S14. ^{1}H NMR spectrum (*δ*, acetone-d6, 600 MHz) of 3′-methylflavanone (**6B**)
Figure S15. ^{1}H NMR spectrum expansion (*δ*, acetone-d6, 600 MHz) of 3′-methylflavanone (**6B**)
Figure S16. ^{13}C NMR spectrum (*δ*, acetone-d6, 151 MHz) of 3′-methylflavanone (**6B**)
Figure S17. ^{13}C NMR spectrum expansion (*δ*, acetone-d6, 151 MHz) of 3′-methylflavanone (**6B**)
Figure S18. COSY contour map – ^{1}H x ^{1}H of 3′-methylflavanone (**6B**)
Figure S19. COSY contour map – ^{1}H x ^{1}H expansion of 3′-methylflavanone (**6B**)
Figure S20. HSQC contour map – ^{1}H x ^{13}C of 3′-methylflavanone (**6B**)
Figure S21. HSQC contour map – ^{1}H x ^{13}C expansion of 3′-methylflavanone (**6B**)
Figure S22. HMBC contour map – ^{1}H x ^{13}C expansion of 3′-methylflavanone (**6B**)
Figure S23. HMBC contour map – ^{1}H x ^{13}C expansion of 3′-methylflavanone (**6B**)
Table S1. The effect of methyl-derivatives of flavanone on the production IL-1β in compared to control in LPS stimulated RAW264.1 cells (n=3). Statistical significance was analysed using Fisher's LSD test. Results marked in red are statistically significant in Fisher's LSD test. Multivariate Tests of Significance ($F = 7.499$, $p < 0.05$).
Table S2. The effect of methyl-derivatives of flavanone on the production IL-6 in compared to control in LPS stimulated RAW264.1 cells (n=3). Statistical significance was analysed using Fisher's LSD test. Results marked in red are statistically significant in Fisher's LSD test. Multivariate Tests of Significance ($F = 7.499$, $p < 0.05$).
Table S3. The effect of methyl-derivatives of flavanone on the production IL-12p40 in compared to control in LPS stimulated RAW264.1 cells (n=3). Statistical significance was analysed using Fisher's LSD test. Results marked in red are statistically significant in Fisher's LSD test. Multivariate Tests of Significance ($F = 7.499$, $p < 0.05$).
Table S4. The effect of methyl-derivatives of flavanone on the production IL-12p70 in compared to control in LPS stimulated RAW264.1 cells (n=3). Statistical significance was analysed using Fisher's LSD test. Results marked in red are statistically significant in Fisher's LSD test. Multivariate Tests of Significance ($F = 7.499$, $p < 0.05$).
Table S5. The effect of methyl-derivatives of flavanone on the production TNF-*α* in compared to control in LPS stimulated RAW264.1 cells (n=3). Statistical significance was analysed using Fisher's LSD test. Results marked in red are statistically significant in Fisher's LSD test. Multivariate Tests of Significance ($F = 7.499$, $p < 0.05$).
Table S6. Average, standard deviations, statistical significance of cytotoxic activity of methyl-derivatives of flavanone. Statistical significance was calculated using t-test (Table S3).
Table S7. Average, standard deviations, statistical significance of chemiluminescence of methyl-derivatives of flavanone. Statistical significance was calculated using t-test (Table S5).
Table S8. The effect of methyl-derivatives of flavanone on the concentration of nitrite in compared to control in LPS stimulated RAW264.1 cells (n=3). Statistical significance was analysed using Fisher's LSD test. Results marked in red are statistically significant in Fisher's LSD test. Multivariate Tests of Significance ($F = 7.499$, $p < 0$).

Figure S1. MS analysis of 2′-hydroxy-3-methylchalcone (**6A**)

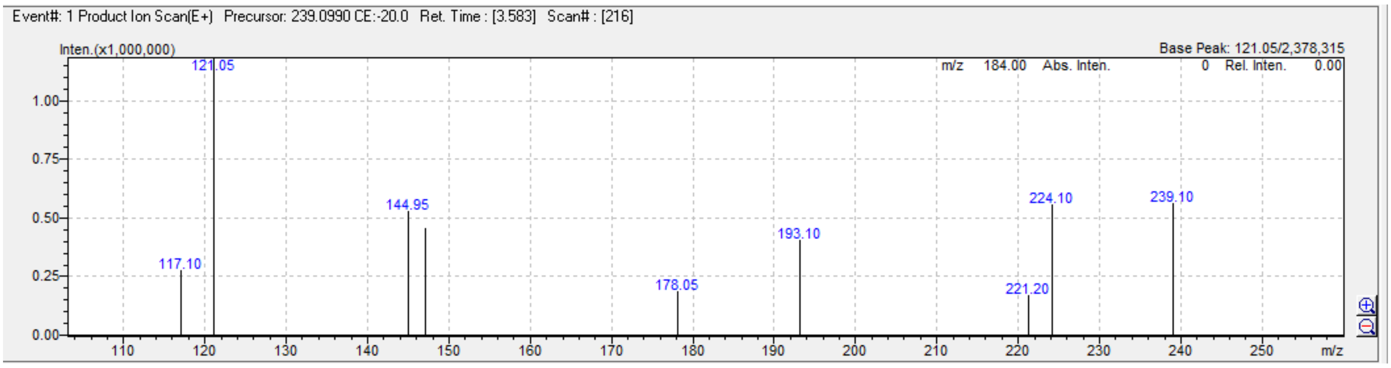

Figure S2. ^{1}H NMR spectrum (δ, acetone-d6, 600 MHz) of 2′-hydroxy-3-methylchalcone (**6A**)

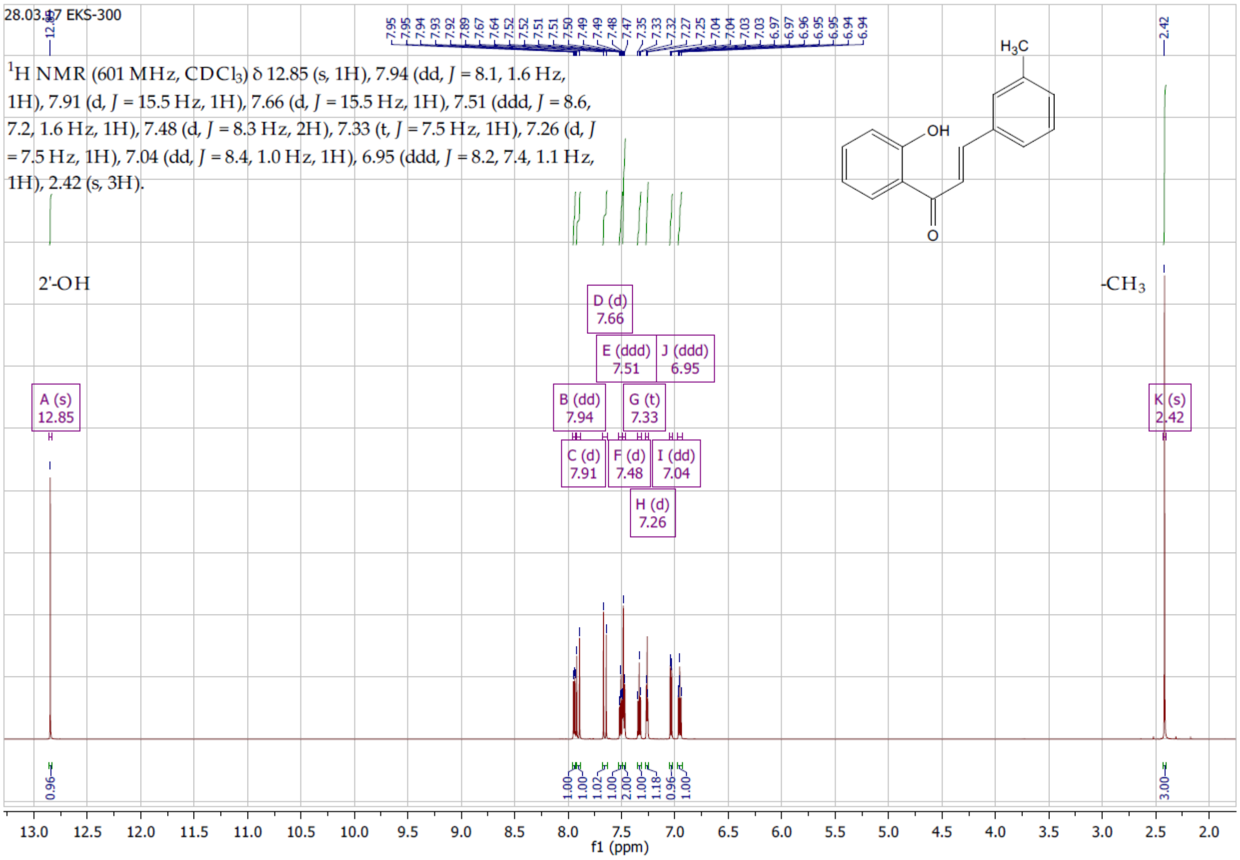

Figure S3 ^{1}H NMR spectrum expansion (δ, acetone-d6, 600 MHz) of 2′-hydroxy-3-methylchalcone (**6A**)

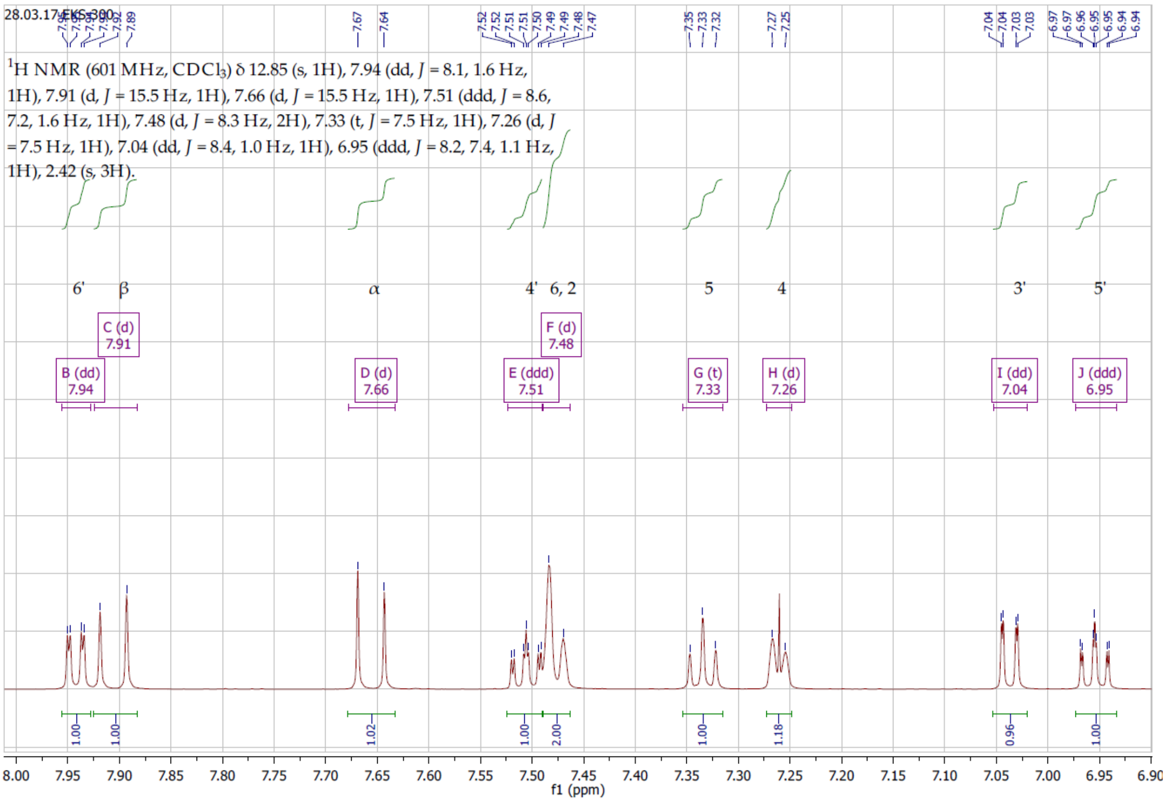

Figure S4. ^{13}C NMR spectrum (δ, acetone-d6, 151 MHz) of 2′-hydroxy-3-methylchalcone (**6A**)

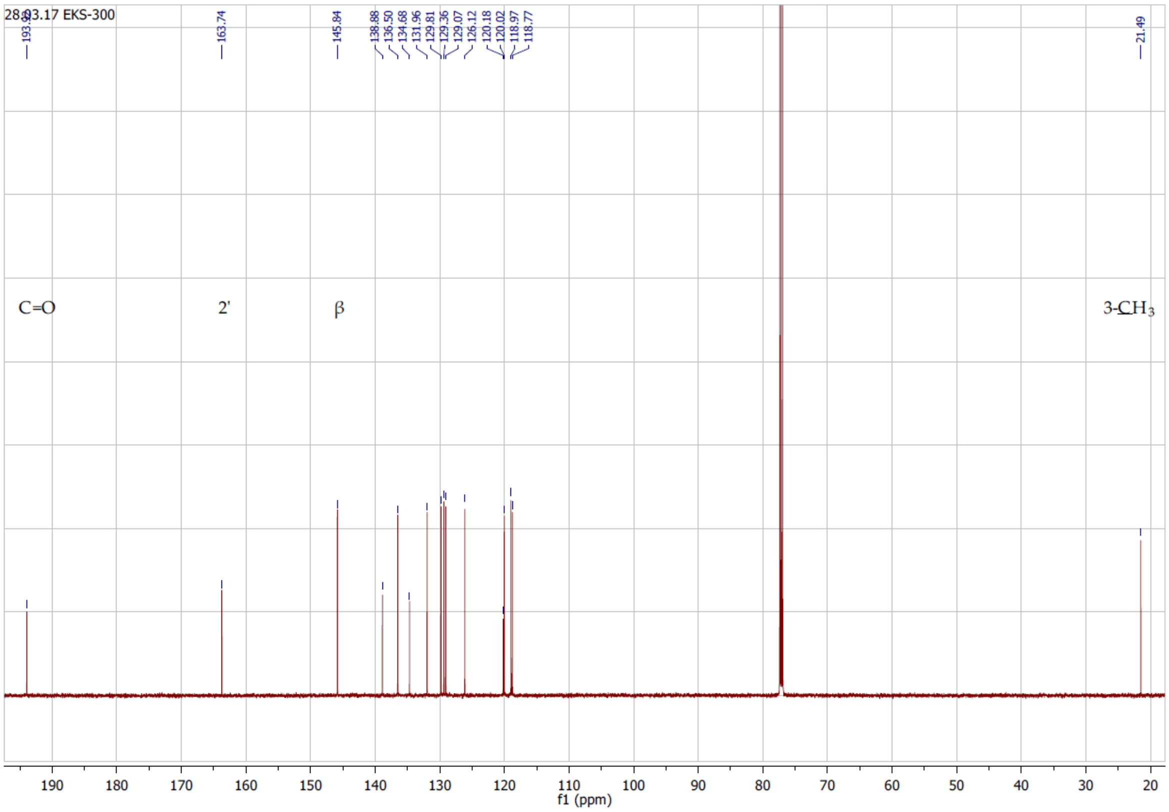

Figure S5. ^{13}C NMR spectrum expansion (δ, acetone-d6, 151 MHz) of 2′-hydroxy-3-methylchalcone (**6A**)

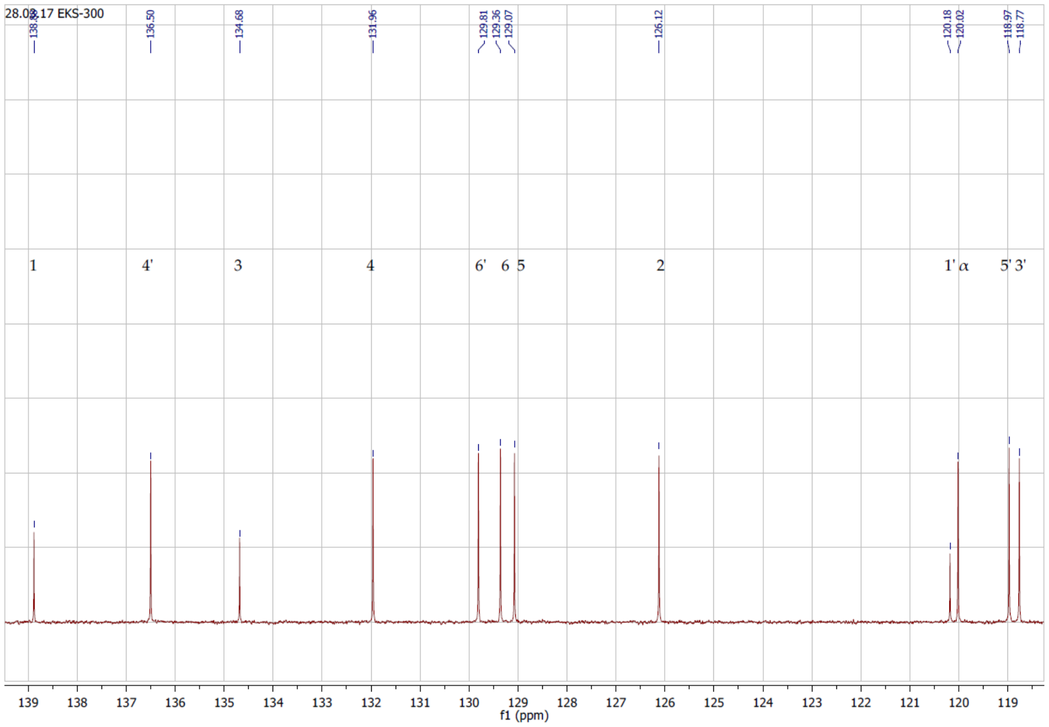

Figure S6. COSY contour map – ^1H x ^1H of 2′-hydroxy-3-methylchalcone (**6A**)

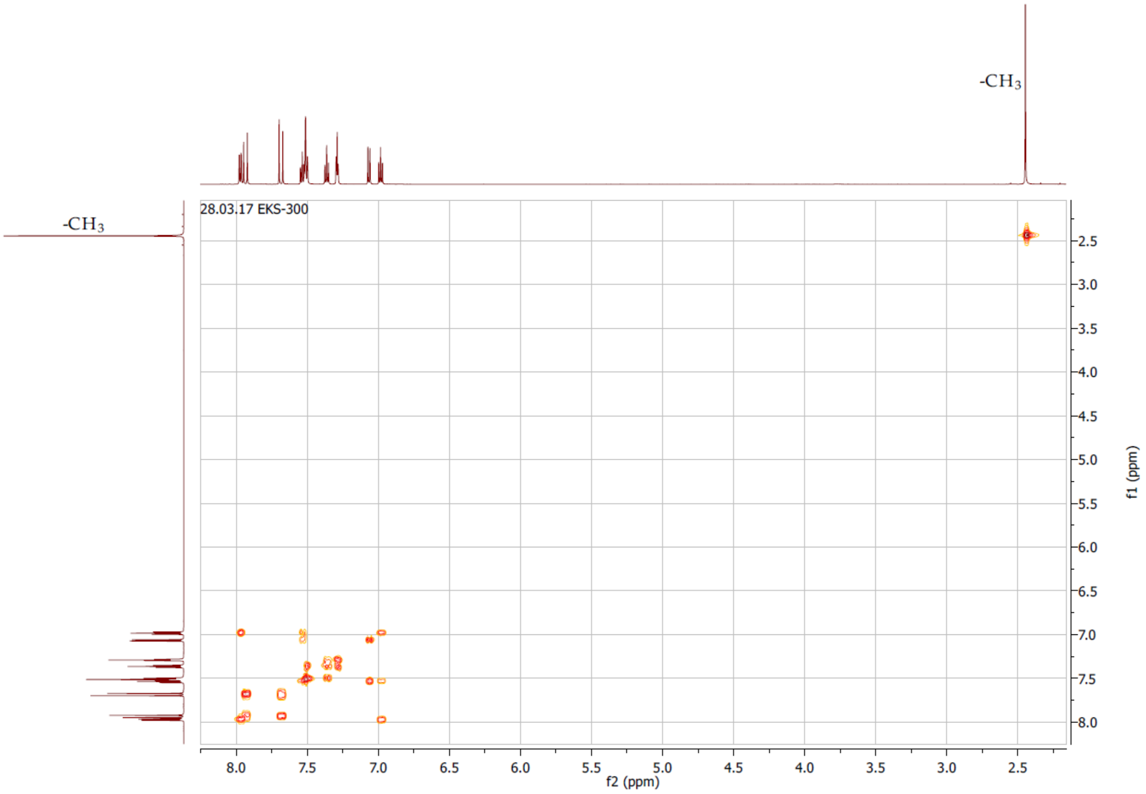

Figure S7. COSY contour map – ^{1}H x ^{1}H expansion of 2′-hydroxy-3-methylchalcone (**6A**)

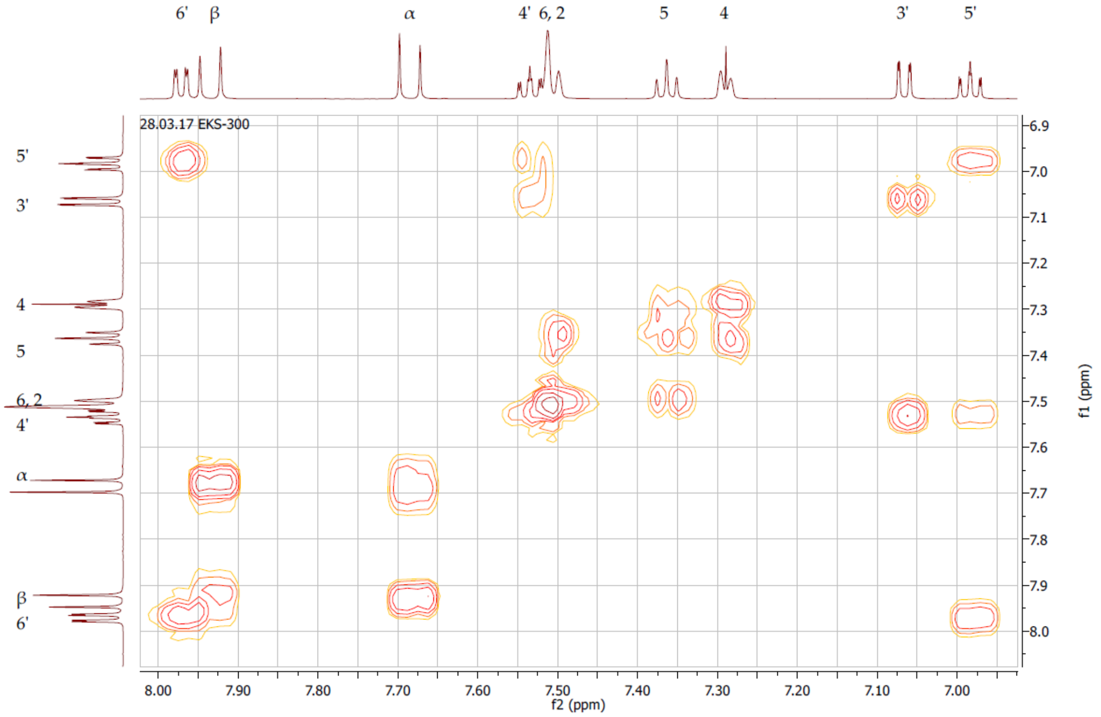

Figure S8. HSQC contour map – ^{1}H x ^{13}C of 2′-hydroxy-3-methylchalcone (**6A**)

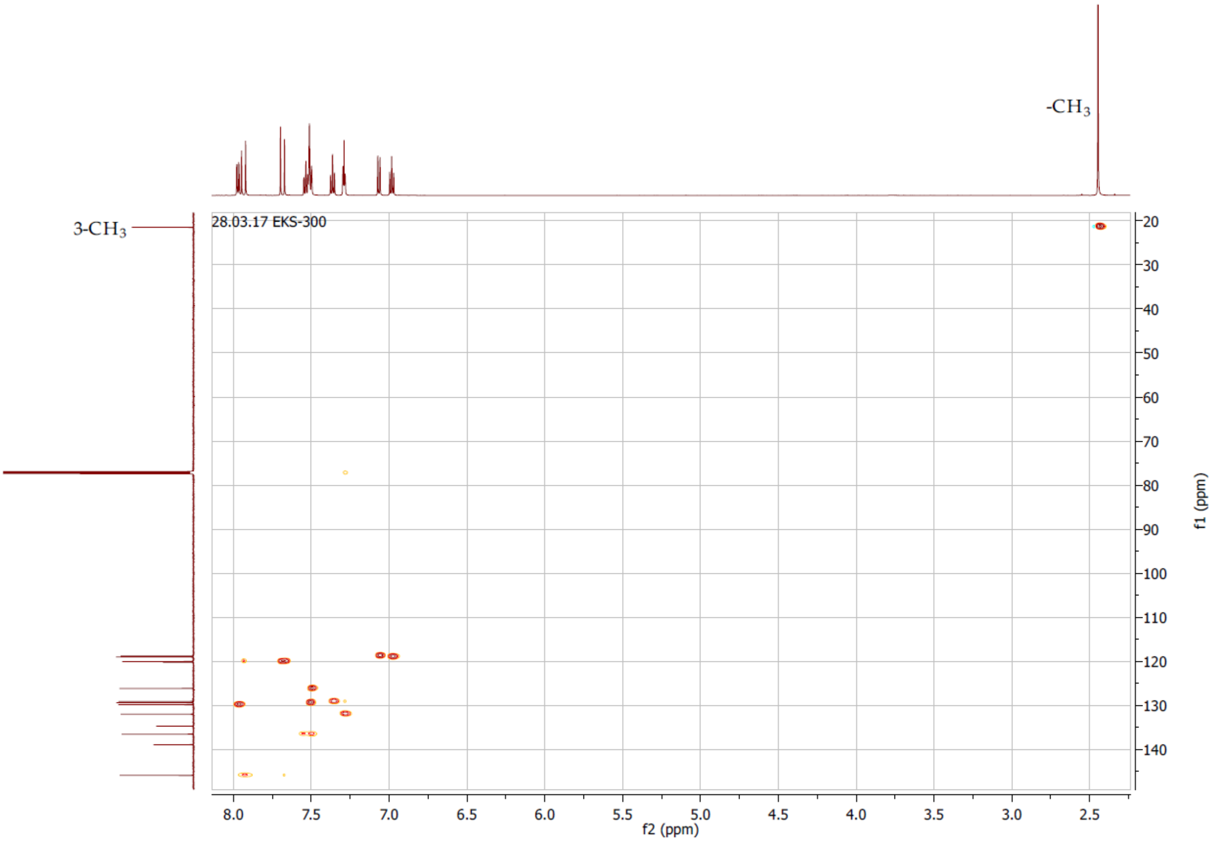

Figure S9. HSQC contour map – ¹H x ¹³C expansion of 2′-hydroxy-3-methylchalcone (**6A**)

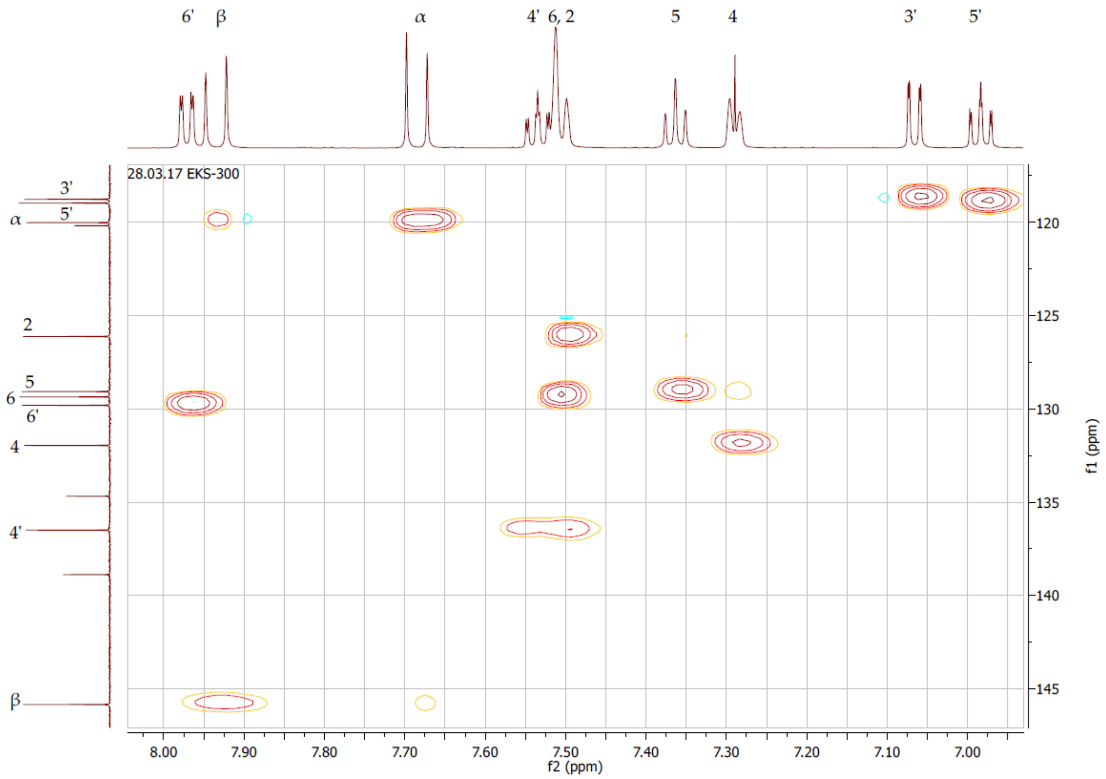

Figure S10. HMBC contour map – ¹H x ¹³C of 2′-hydroxy-3-methylchalcone (**6A**)

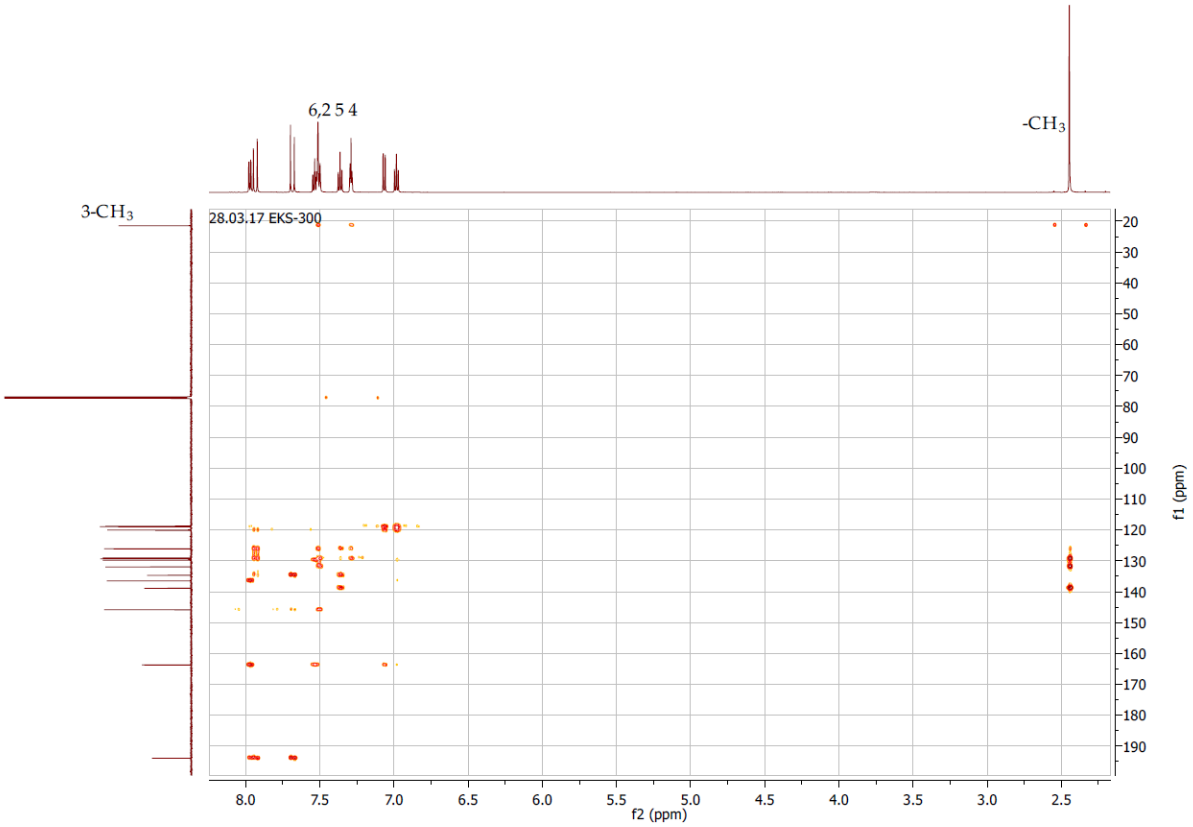

Figure S11. HMBC contour map – ^{1}H x ^{13}C expansion of 2′-hydroxy-3-methylchalcone (**6A**)

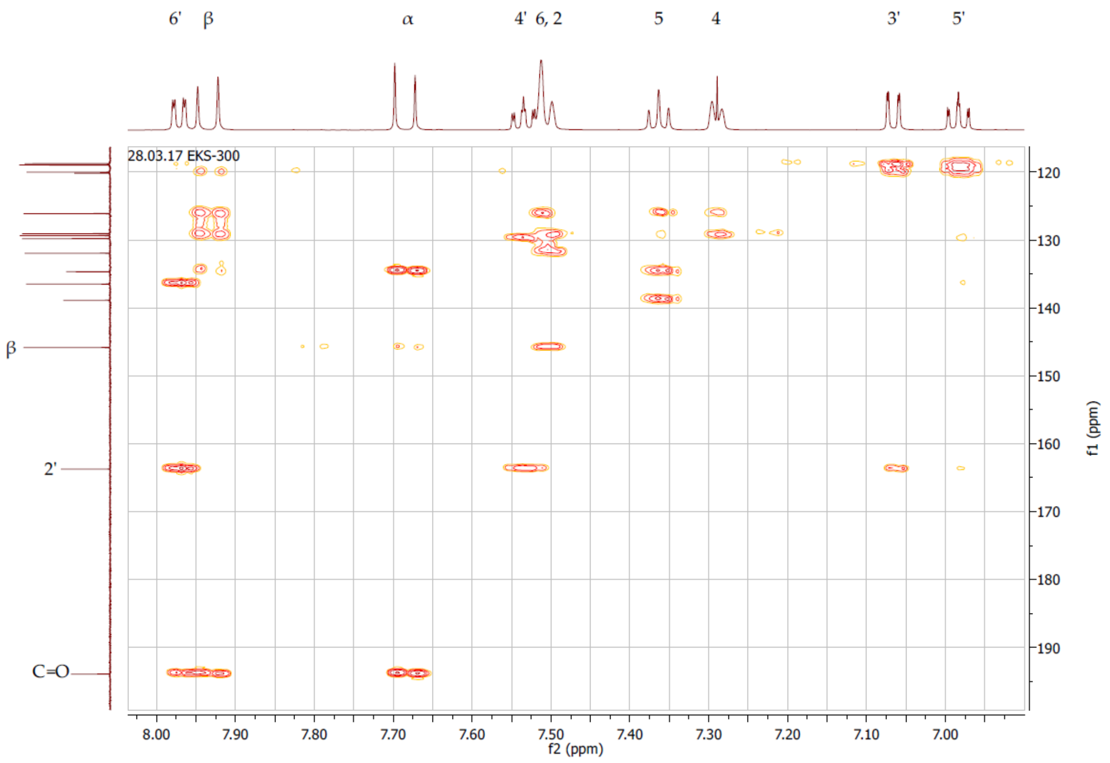

Figure S12. HMBC contour map – ^{1}H x ^{13}C expansion of 2′-hydroxy-3-methylchalcone (**6A**)

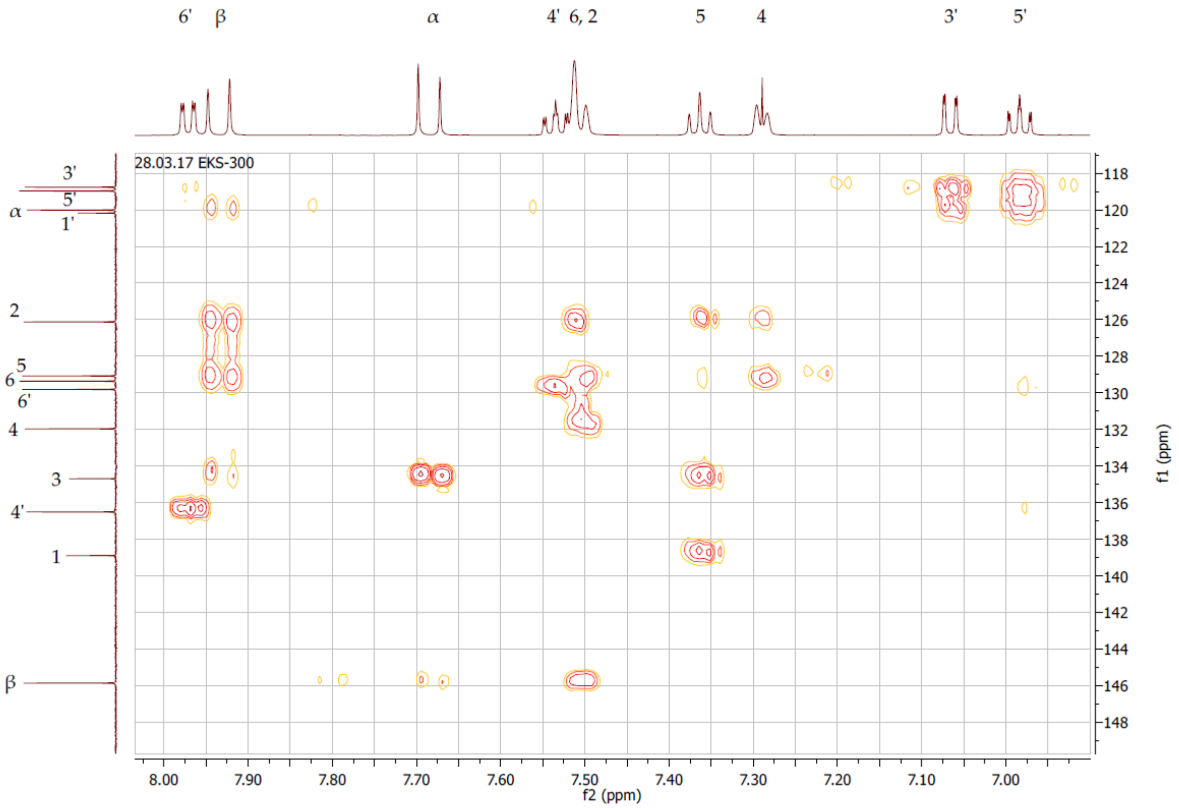

Figure S13. MS analysis of 3′-methylflavanone (**6B**)

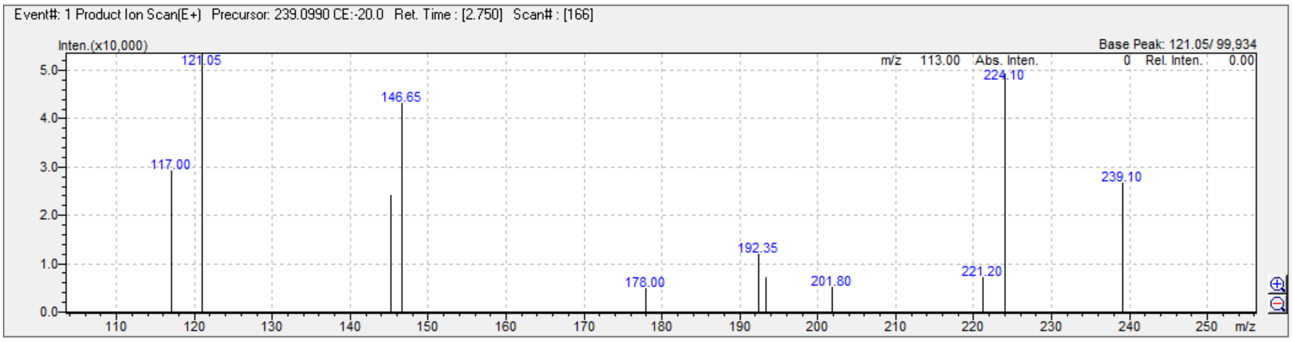

Figure S14. ^{1}H NMR spectrum (δ, acetone-d6, 600 MHz) of 3′-methylflavanone (**6B**)

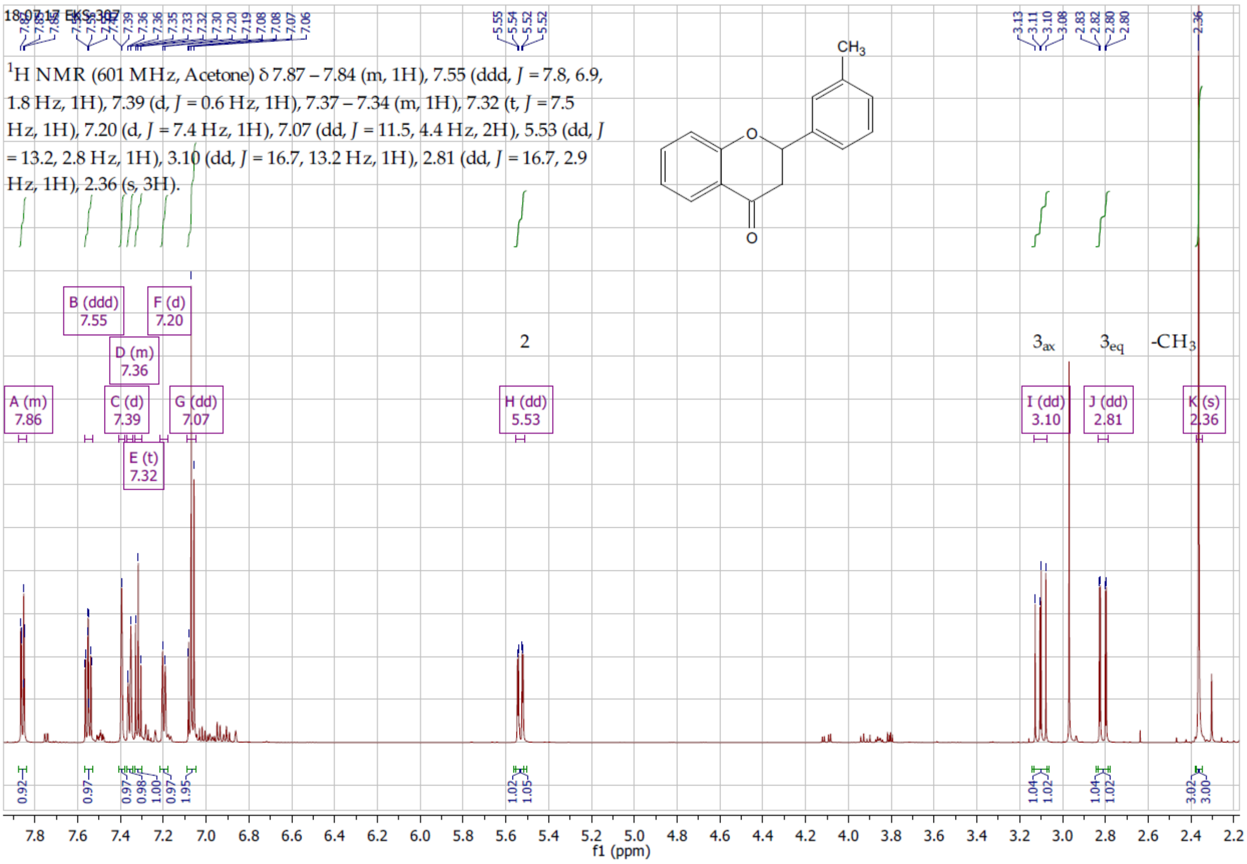

Figure S15. ^{1}H NMR spectrum expansion (δ, acetone-d6, 600 MHz) of 3′-methylflavanone (**6B**)

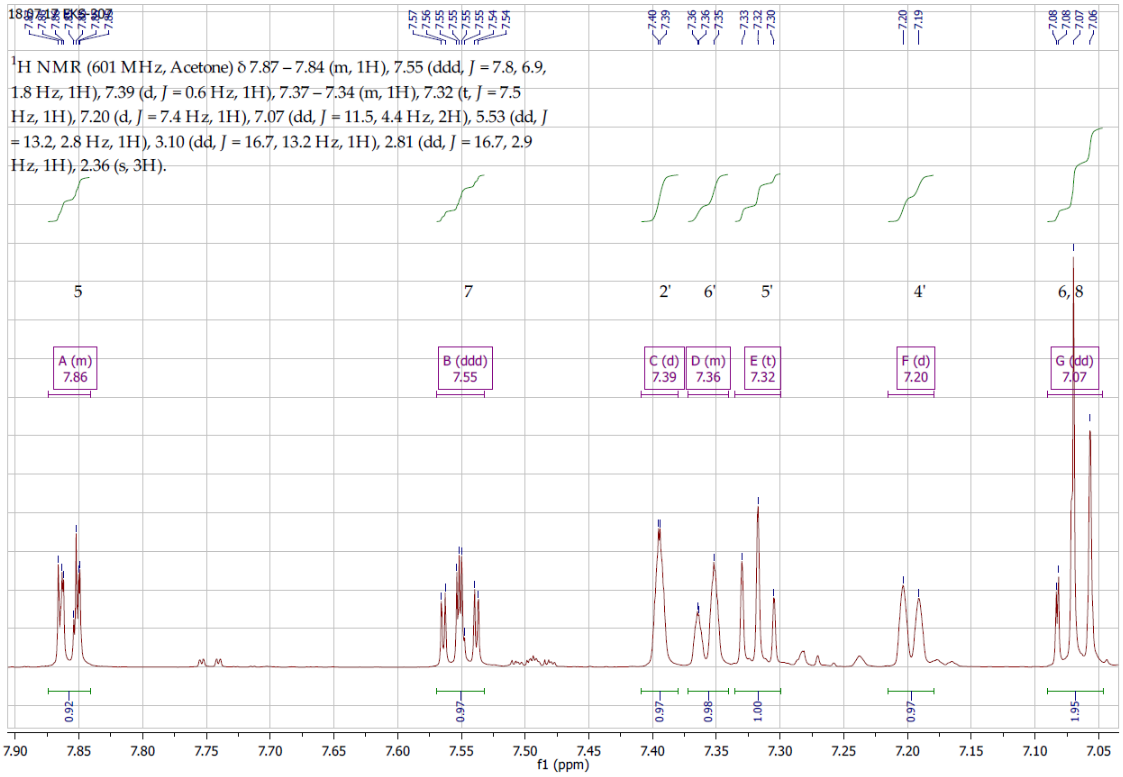

Figure S16. ^{13}C NMR spectrum (δ, acetone-d6, 151 MHz) of 3′-methylflavanone (**6B**)

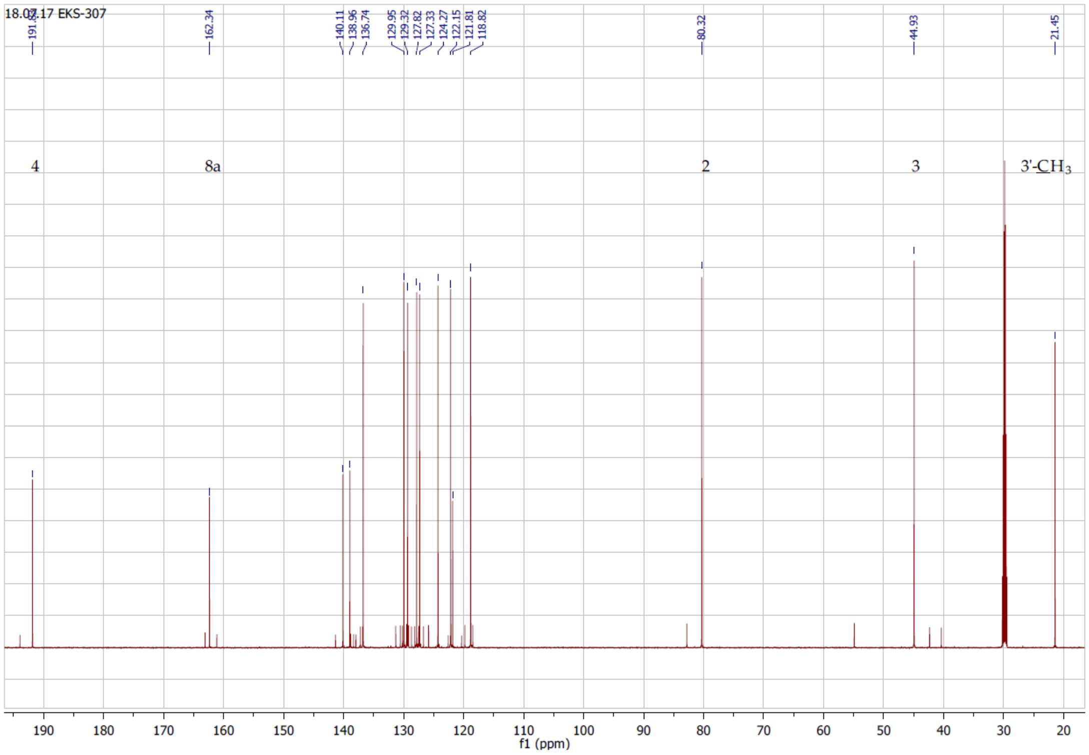

Figure S17. ^{13}C NMR spectrum expansion (δ, acetone-d6, 151 MHz) of 3′-methylflavanone (**6B**)

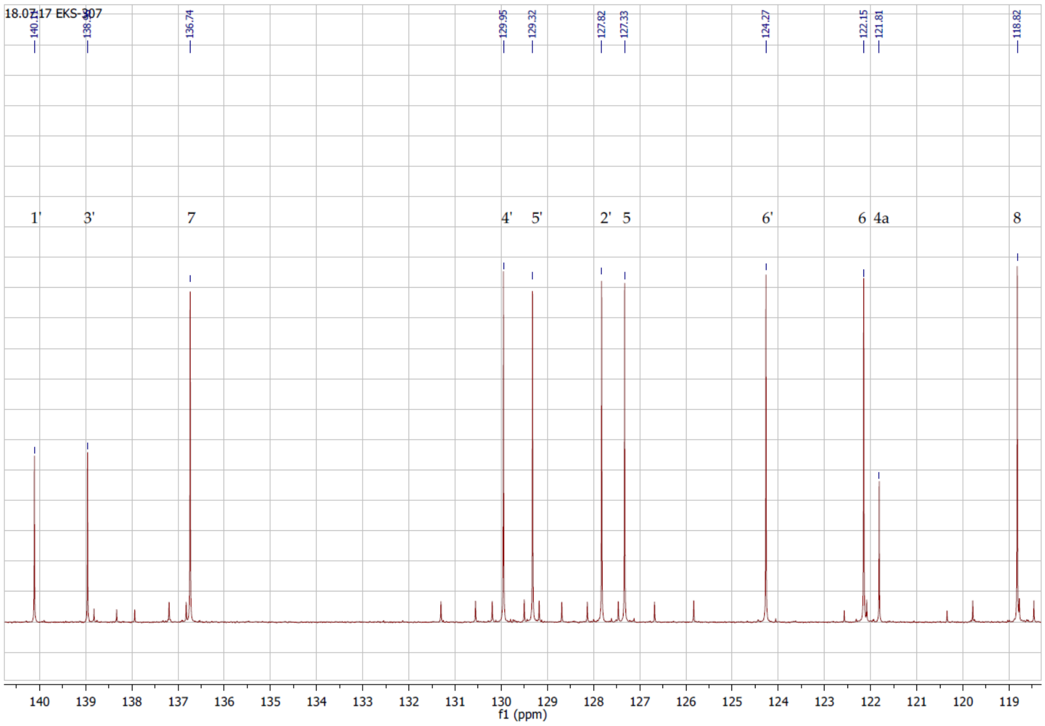

Figure S18. COSY contour map – ^{1}H x ^{1}H of 3′-methylflavanone (**6B**)

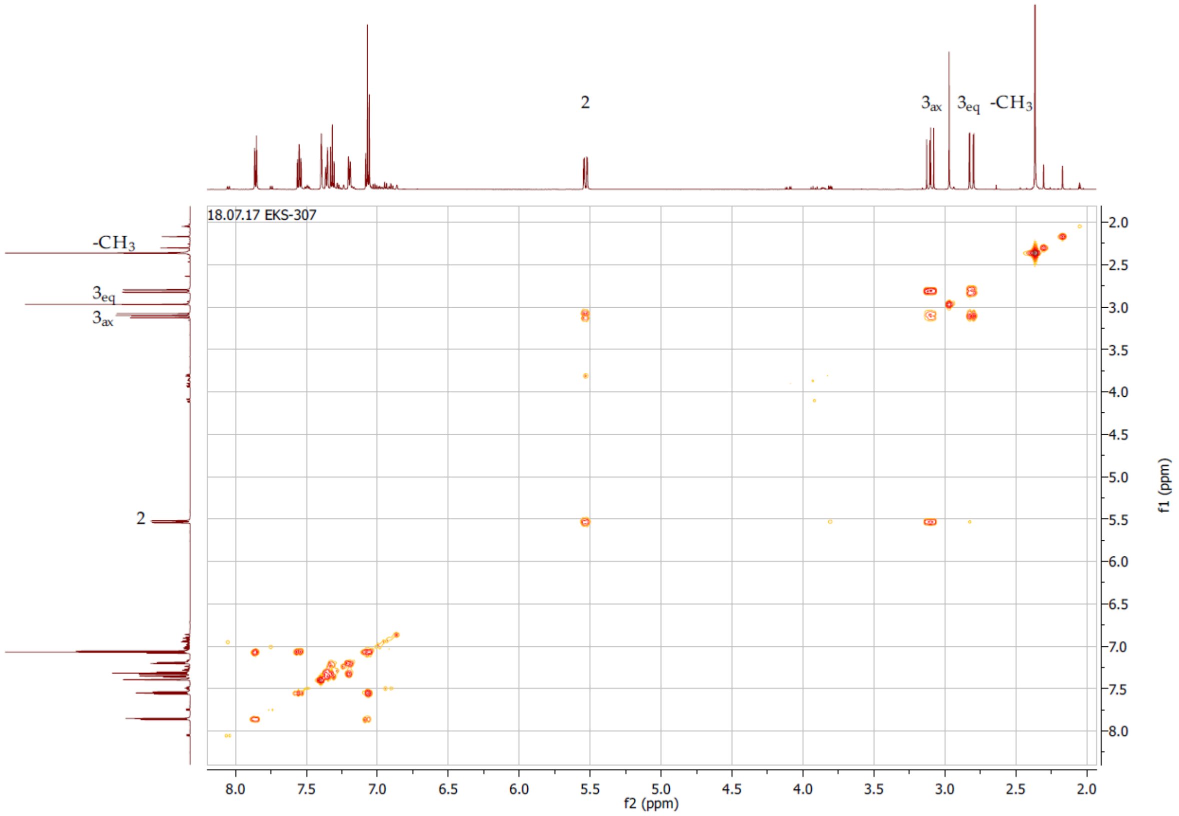

Figure S19. COSY contour map – ¹H x ¹H expansion of 3′-methylflavanone (**6B**)

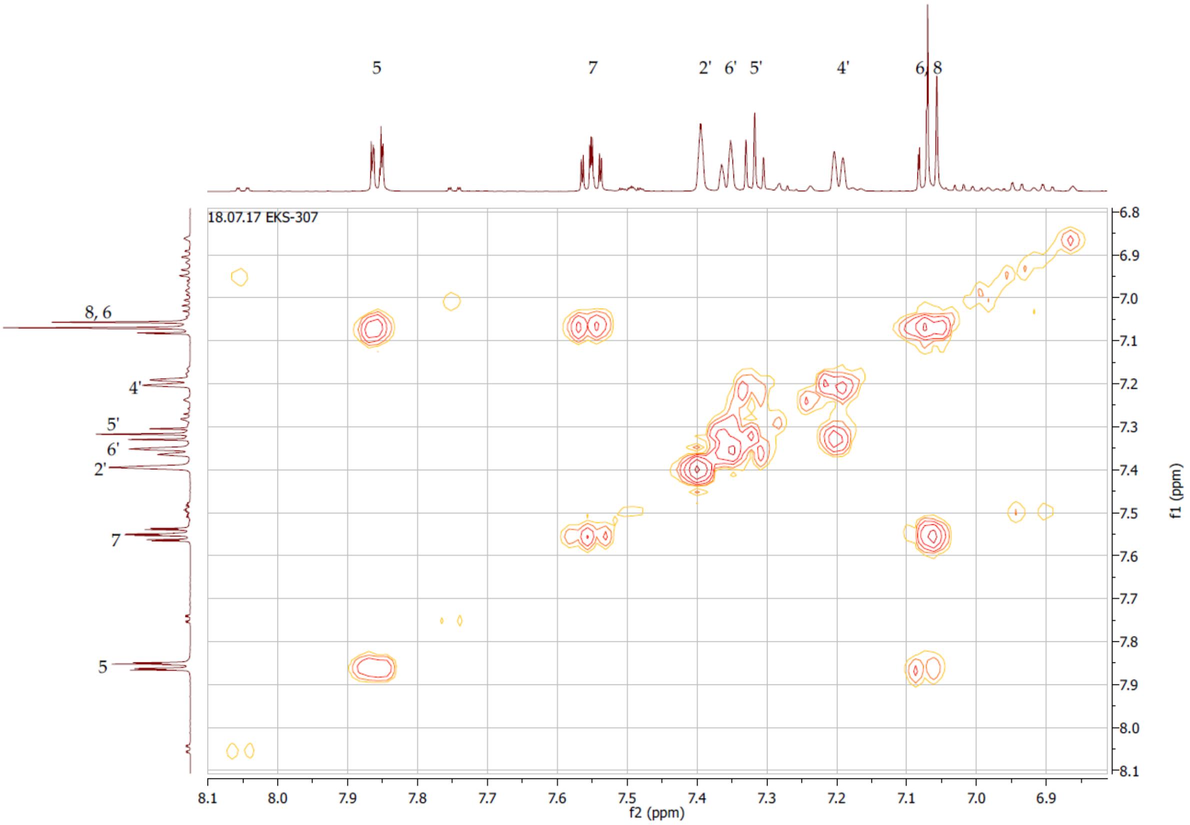

Figure S20. HSQC contour map – ¹H x ¹³C of 3′-methylflavanone (**6B**)

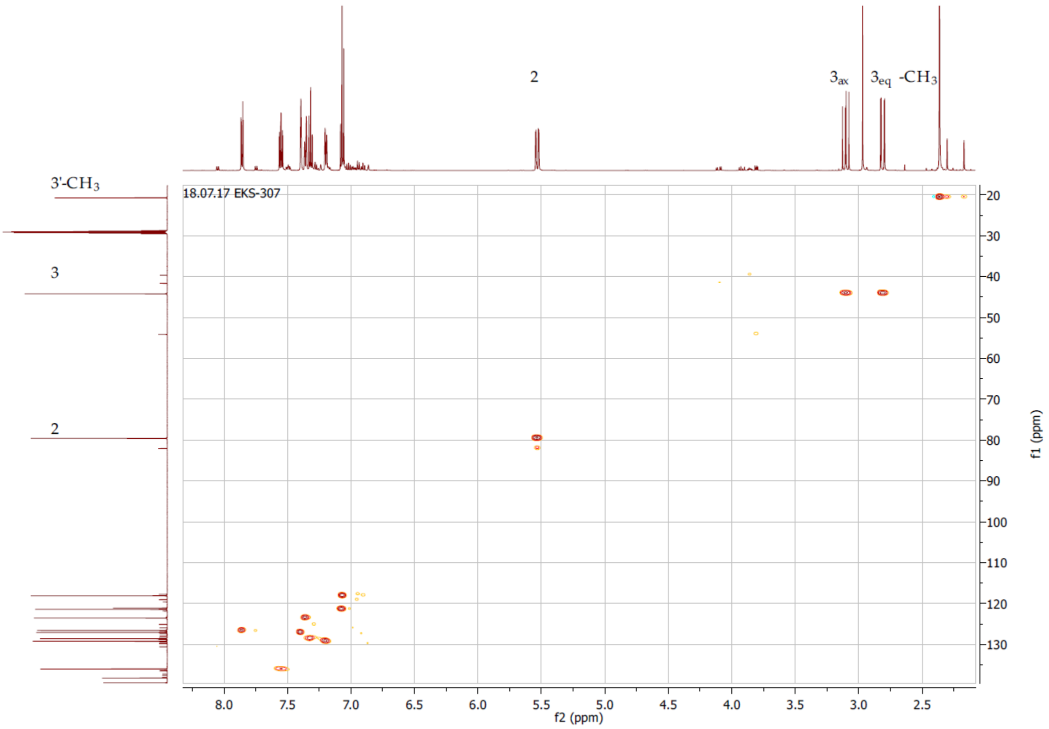

Figure S21. HSQC contour map – ^{1}H x ^{13}C expansion of 3′-methylflavanone (**6B**)

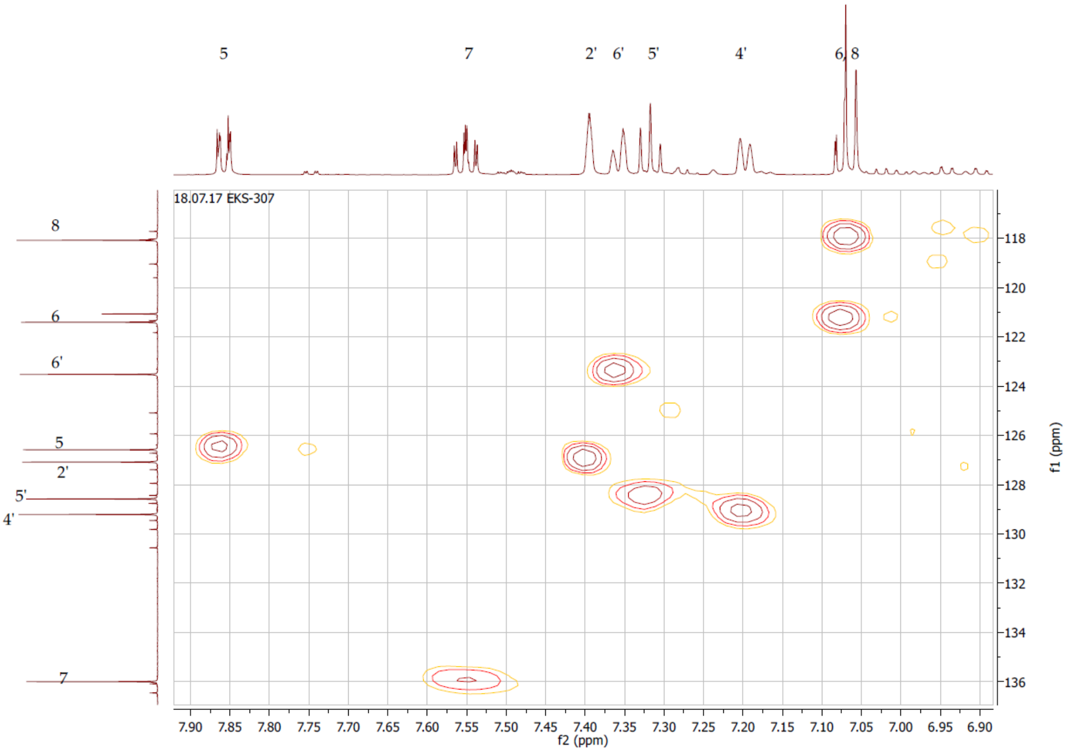

Figure S22. HMBC contour map – ^{1}H x ^{13}C expansion of 3′-methylflavanone (**6B**)

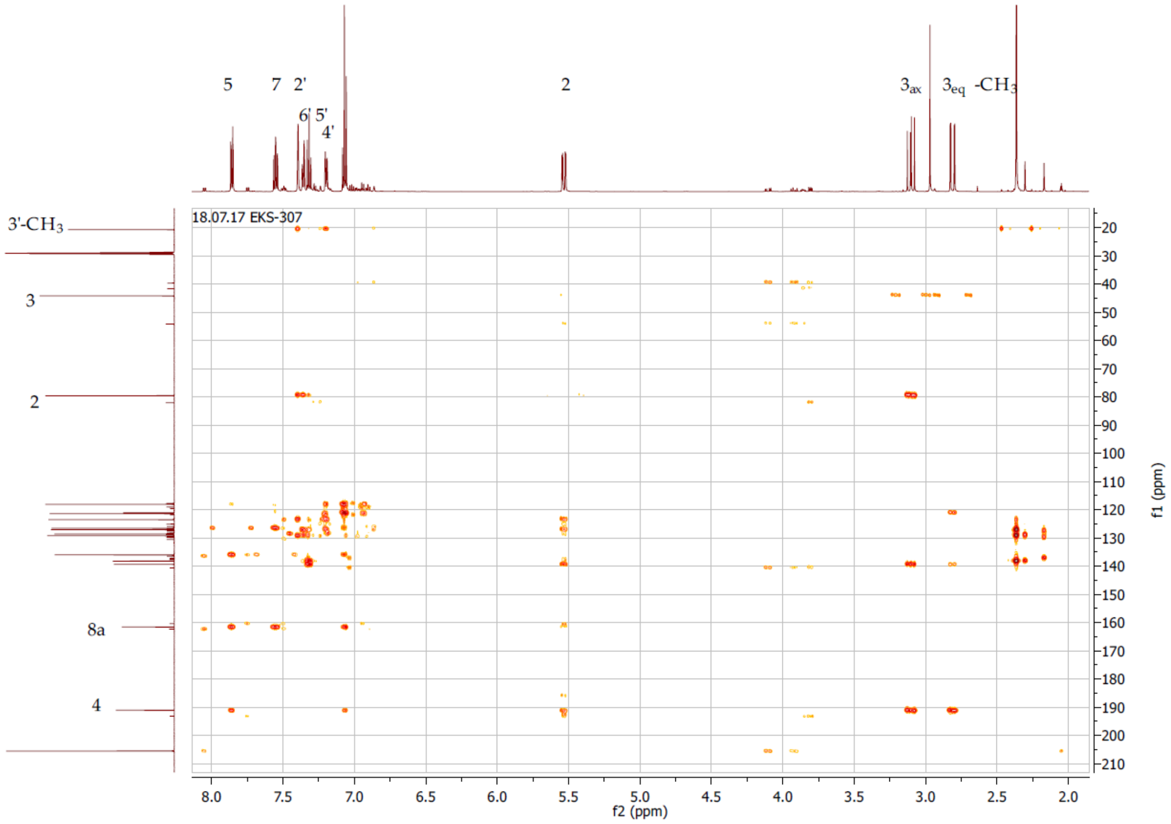

Figure S23. HMBC contour map – ^{1}H x ^{13}C expansion of 3′-methylflavanone (**6B**)

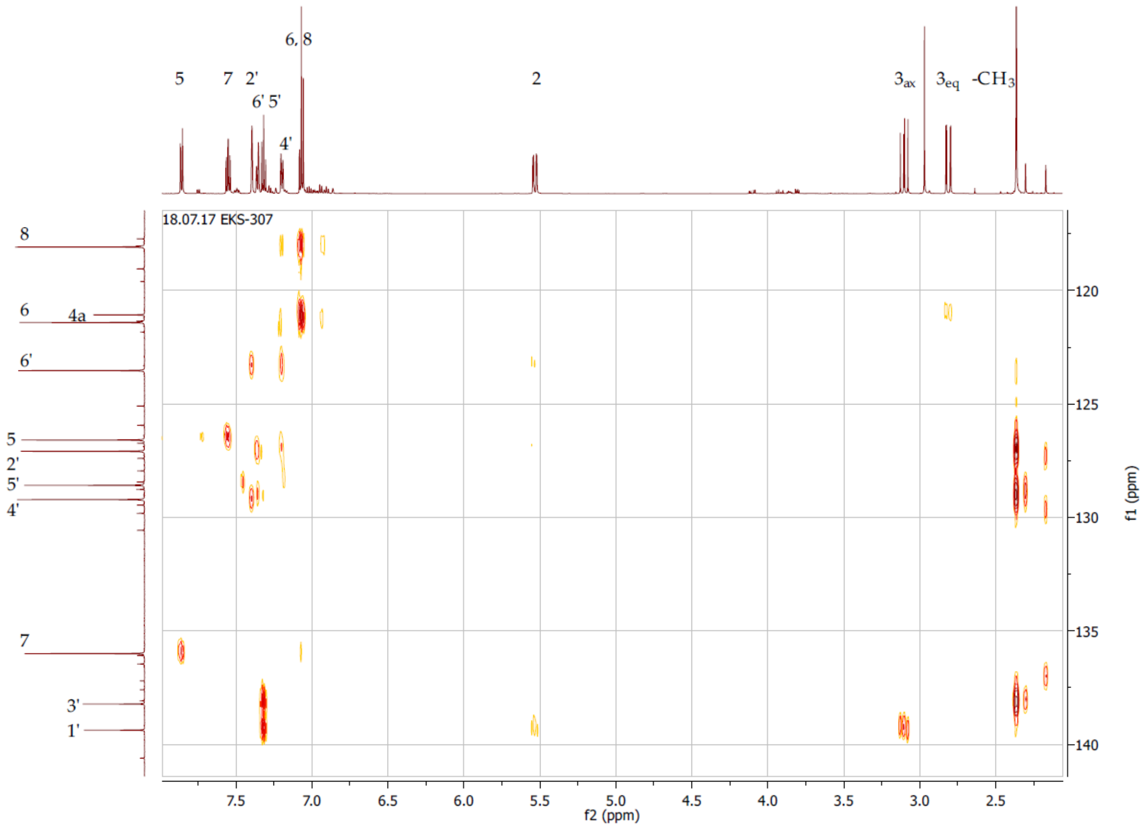

Table S1. The effect of methyl-derivatives of flavanone on the production IL-1β in compared to control in LPS stimulated RAW264.1 cells (n=3). Statistical significance was analysed using Fisher's LSD test. Results marked in red are statistically significant in Fisher's LSD test. Multivariate Tests of Significance (F = 7.499, p < 0.05).

Multivariate Tests of Significance
Sigma-restricted parameterization
Effective hypothesis decomposition

Effect	Test	Value	F	Effect	Error	p
Intercept	Wilks	0.001581	2273.118	5	18.00000	0.000000
sample	Wilks	0.000462	7.499	50	85.45684	0.000000

LSD test; variable IL-1ß (sta-zabrze)
Probabilities for Post Hoc Tests
Error: Between MS = 6,1378, df = 22,000

Cell No.	sample	{1}	{2}	{3}	{4}	{5}	{6}	{7}	{8}	{9}	{10}	{11}
1	K DMSO + LPS		0.880882	0.318805	0.009652	0.006865	0.757877	0.963008	0.032619	0.612231	0.875184	0.538352
2	5B 1µM + LPS	0.880882		0.253881	0.006813	0.004823	0.647391	0.917569	0.044695	0.720358	0.994230	0.640541
3	5B 20µM + LPS	0.318805	0.253881		0.083296	0.062462	0.486448	0.297571	0.003257	0.139229	0.251016	0.114164
4	6B 1µM + LPS	0.009652	0.006813	0.083296		0.883452	0.019403	0.008671	0.000040	0.002907	0.006699	0.002231
5	6B 20µM + LPS	0.006865	0.004823	0.062462	0.883452		0.013973	0.006158	0.000028	0.002040	0.004741	0.001562
6	7B 1µM + LPS	0.757877	0.647391	0.486448	0.019403	0.013973		0.722984	0.016612	0.417480	0.642240	0.358826
7	7B 20µM + LPS	0.963008	0.917569	0.297571	0.008671	0.006158	0.722984		0.035993	0.644880	0.911834	0.569024
8	8B 1µM + LPS	0.032619	0.044695	0.003257	0.000040	0.000028	0.016612	0.035993		0.091209	0.045369	0.112020
9	8B 20µM + LPS	0.612231	0.720358	0.139229	0.002907	0.002040	0.417480	0.644880	0.091209		0.725753	0.912729
10	flawanon 1µM + LPS	0.875184	0.994230	0.251016	0.006699	0.004741	0.642240	0.911834	0.045369	0.725753		0.645685
11	flawanon 20µM + LPS	0.538352	0.640541	0.114164	0.002231	0.001562	0.358826	0.569024	0.112020	0.912729	0.645685	

Table S2. The effect of methyl-derivatives of flavanone on the production IL-6 in compared to control in LPS stimulated RAW264.1 cells (n=3). Statistical significance was analysed using Fisher's LSD test. Results marked in red are statistically significant in Fisher's LSD test. Multivariate Tests of Significance ($F = 7.499$, $p < 0.05$).

LSD test; variable **IL-6**

Probabilities for Post Hoc Tests

Error: Between MS = 25771,, df = 22,000

	sample	{1}	{2}	{3}	{4}	{5}	{6}	{7}	{8}	{9}	{10}	{11}
1	K DMSO + LPS		0.014881	0.000133	0.018428	0.000066	0.002296	0.804608	0.001515	0.735847	0.253626	0.005065
2	5B 1µM + LPS	0.014881		0.060733	0.923936	0.033538	0.000004	0.008447	0.000003	0.006845	0.000947	0.000009
3	5B 20µM + LPS	0.000133	0.060733		0.050051	0.774094	0.000000	0.000072	0.000000	0.000058	0.000008	0.000000
4	6B 1µM + LPS	0.018428	0.923936	0.050051		0.027329	0.000005	0.010529	0.000003	0.008550	0.001197	0.000011
5	6B 20µM + LPS	0.000066	0.033538	0.774094	0.027329		0.000000	0.000036	0.000000	0.000029	0.000004	0.000000
6	7B 1µM + LPS	0.002296	0.000004	0.000000	0.000005	0.000000		0.004162	0.864110	0.005156	0.032999	0.741529
7	7B 20µM + LPS	0.804608	0.008447	0.000072	0.010529	0.000036	0.004162		0.002761	0.928103	0.366591	0.009041
8	8B 1µM + LPS	0.001515	0.000003	0.000000	0.000003	0.000000	0.864110	0.002761		0.003430	0.022795	0.617078
9	8B 20µM + LPS	0.735847	0.006845	0.000058	0.008550	0.000029	0.005156	0.928103	0.003430		0.415118	0.011128
10	flawanon 1µM + LPS	0.253626	0.000947	0.000008	0.001197	0.000004	0.032999	0.366591	0.022795	0.415118		0.065170
11	flawanon 20µM + LPS	0.005065	0.000009	0.000000	0.000011	0.000000	0.741529	0.009041	0.617078	0.011128	0.065170	

Table S3. The effect of methyl-derivatives of flavanone on the production IL-6 in compared to control in LPS stimulated RAW264.1 cells (n=3). Statistical significance was analysed using Fisher's LSD test. Results marked in red are statistically significant in Fisher's LSD test. Multivariate Tests of Significance ($F = 7.499$, $p < 0.05$).

LSD test; variable **IL-12p40**

Probabilities for Post Hoc Tests

Error: Between MS = 10,535, df = 22,000

	sample	{1}	{2}	{3}	{4}	{5}	{6}	{7}	{8}	{9}	{10}	{11}
1	K DMSO + LPS		0.001031	0.000307	0.038380	0.010886	0.065044	0.232104	0.362711	0.365265	0.205639	0.946897
2	5B 1µM + LPS	0.001031		0.624142	0.129111	0.328996	0.079665	0.018222	0.009310	0.009203	0.021488	0.000875
3	5B 20µM + LPS	0.000307	0.624142		0.050010	0.149056	0.029096	0.005902	0.002917	0.002882	0.007030	0.000260
4	6B 1µM + LPS	0.038380	0.129111	0.050010		0.568785	0.796677	0.340662	0.216194	0.214448	0.378589	0.033339
5	6B 20µM + LPS	0.010886	0.328996	0.149056	0.568785		0.410318	0.134813	0.077516	0.076769	0.153812	0.009340
6	7B 1µM + LPS	0.065044	0.079665	0.029096	0.796677	0.410318		0.483236	0.322293	0.319948	0.530175	0.056918
7	7B 20µM + LPS	0.232104	0.018222	0.005902	0.340662	0.134813	0.483236		0.767478	0.763693	0.940615	0.208327
8	8B 1µM + LPS	0.362711	0.009310	0.002917	0.216194	0.077516	0.322293	0.767478		0.996031	0.711464	0.329661
9	8B 20µM + LPS	0.365265	0.009203	0.002882	0.214448	0.076769	0.319948	0.763693	0.996031		0.707779	0.332055
10	flawanon 1µM + LPS	0.205639	0.021488	0.007030	0.378589	0.153812	0.530175	0.940615	0.711464	0.707779		0.184018
11	flawanon 20µM + LPS	0.946897	0.000875	0.000260	0.033339	0.009340	0.056918	0.208327	0.329661	0.332055	0.184018	

Table S4. The effect of methyl-derivatives of flavanone on the production IL-12p70 in compared to control in LPS stimulated RAW264.1 cells (n=3). Statistical significance was analysed using Fisher's LSD test. Results marked in red are statistically significant in Fisher's LSD test. Multivariate Tests of Significance ($F = 7.499$, $p < 0.05$).

LSD test; variable **IL-12p70**

Probabilities for Post Hoc Tests

Error: Between MS = 15,184, df = 22,000

Cell No.	sample	{1}	{2}	{3}	{4}	{5}	{6}	{7}	{8}	{9}	{10}	{11}
1	K DMSO + LPS		0.003850	0.000410	0.000050	0.000010	0.264156	0.005071	0.121969	0.000408	0.111074	0.570728
2	5B 1µM + LPS	0.003850		0.363357	0.086633	0.021724	0.000241	0.000002	0.000078	0.000000	0.000069	0.014484
3	5B 20µM + LPS	0.000410	0.363357		0.396171	0.137294	0.000025	0.000000	0.000008	0.000000	0.000007	0.001660
4	6B 1µM + LPS	0.000050	0.086633	0.396171		0.505590	0.000003	0.000000	0.000001	0.000000	0.000001	0.000202
5	6B 20µM + LPS	0.000010	0.021724	0.137294	0.505590		0.000001	0.000000	0.000000	0.000000	0.000000	0.000039
6	7B 1µM + LPS	0.264156	0.000241	0.000025	0.000003	0.000001		0.061933	0.648135	0.006379	0.612198	0.099198
7	7B 20µM + LPS	0.005071	0.000002	0.000000	0.000000	0.000000	0.061933		0.146748	0.306270	0.160439	0.001286
8	8B 1µM + LPS	0.121969	0.000078	0.000008	0.000001	0.000000	0.648135	0.146748		0.018187	0.959332	0.039895
9	8B 20µM + LPS	0.000408	0.000000	0.000000	0.000000	0.000000	0.006379	0.306270	0.018187		0.020364	0.000100
10	flawanon 1µM + LPS	0.111074	0.000069	0.000007	0.000001	0.000000	0.612198	0.160439	0.959332	0.020364		0.035837
11	flawanon 20µM + LPS	0.570728	0.014484	0.001660	0.000202	0.000039	0.099198	0.001286	0.039895	0.000100	0.035837	

Table S5. The effect of methyl-derivatives of flavanone on the production TNF-*α* in compared to control in LPS stimulated RAW264.1 cells (n=3). Statistical significance was analysed using Fisher's LSD test. Results marked in red are statistically significant in Fisher's LSD test. Multivariate Tests of Significance ($F = 7.499$, $p < 0.05$).

LSD test; variable **TNF-α**

Probabilities for Post Hoc Tests

Error: Between MS = 3334E4, df = 22,000

	sample	{1}	{2}	{3}	{4}	{5}	{6}	{7}	{8}	{9}	{10}	{11}
1	K DMSO + LPS		0.000296	0.000095	0.826990	0.974889	0.927038	0.373381	0.531949	0.069246	0.571884	0.008844
2	5B 1µM + LPS	0.000296		0.646828	0.000172	0.000274	0.000236	0.000032	0.000063	0.000003	0.000073	0.000000
3	5B 20µM + LPS	0.000095	0.646828		0.000056	0.000088	0.000076	0.000011	0.000021	0.000001	0.000024	0.000000
4	6B 1µM + LPS	0.826990	0.000172	0.000056		0.851557	0.898877	0.498972	0.682982	0.105365	0.727688	0.014581
5	6B 20µM + LPS	0.974889	0.000274	0.000088	0.851557		0.952075	0.390062	0.552539	0.073666	0.593252	0.009512
6	7B 1µM + LPS	0.927038	0.000236	0.000076	0.898877	0.952075		0.423234	0.592983	0.082795	0.635104	0.010921
7	7B 20µM + LPS	0.373381	0.000032	0.000011	0.498972	0.390062	0.423234		0.786939	0.327509	0.740952	0.062300
8	8B 1µM + LPS	0.531949	0.000063	0.000021	0.682982	0.552539	0.592983	0.786939		0.215599	0.951763	0.035704
9	8B 20µM + LPS	0.069246	0.000003	0.000001	0.105365	0.073666	0.082795	0.327509	0.215599		0.195135	0.346267
10	flawanon 1µM + LPS	0.571884	0.000073	0.000024	0.727688	0.593252	0.635104	0.740952	0.951763	0.195135		0.031395
11	flawanon 20µM + LPS	0.008844	0.000000	0.000000	0.014581	0.009512	0.010921	0.062300	0.035704	0.346267	0.031395	

Table S6. Average, standard deviations, statistical significance of cytotoxic activity of methyl-derivatives of flavanone. Statistical significance was calculated using t-test (Table S3).

**Sample**	**RAW264.7 cell viability [%]**		
	**AVG**	**SD**	***p***
Control	97.83	3.14	
5B 1μM	111.04	12.65	0.114254581
5B 10μM	97.93	4.94	0.268060958
5B 20μM	92.17	8.00	0.09701206
6B 1μM	92.47	1.93	0.182880144
6B 10μM	96.81	2.26	0.065944168
6B 20μM	89.21	5.46	0.102198598
7B 1μM	91.32	1.82	0.001728865
7B 10μM	92.95	2.20	0.005413282
7B 20μM	89.71	2.74	0.006185175
8B 1μM	94.67	5.34	0.140268954
8B 10μM	90.45	2.85	0.002481306
8B 20μM	88.45	3.41	0.002088066
Flavanone 1μM	94.46	2.78	0.020122714
Flavanone 10μM	97.94	0.87	0.124969323
Flavanone 20μM	97.82	3.10	0.287500787

Table S7. Average, standard deviations, statistical significance of chemiluminescence of methyl-derivatives of flavanone. Statistical significance was calculated using t-test (Table S5).

**Sample**	**RAW264.7 cell viability [%]**		
	**AVG**	**SD**	***p***
5B 1µM	107.16	9.42	0.604869355
5B 5µM	102.45	5.02	0.006617773
5B 10µM	65.54	5.15	0.012756791
5B 50µM	28.50	3.16	0.026308649
6B 1µM	94.99	11.99	0.093523114
6B 5µM	89.21	9.56	0.003180751
6B 10µM	58.68	3.73	0.000482798
6B 50µM	18.65	3.41	0.000382919
7B 1µM	99.21	11.79	0.192650358
7B 5µM	97.11	11.36	0.01929824
7B 10µM	68.47	3.39	0.010000
7B 50µM	14.85	2.18	0.000205425
8B 1µM	127.34	7.14	0.063302521
8B 5µM	119.74	5.98	0.52493694
8B 10µM	76.15	6.02	0.538279569
8B 50µM	34.18	5.47	0.562122327
Flavanone 1µM	111.36	12.34	
Flavanone 5µM	123.30	6.87	
Flavanone 10µM	78.80	3.43	
Flavanone 50µM	36.35	4.63	

Table S8. The effect of methyl-derivatives of flavanone on the concentration of nitrite in compared to control in LPS stimulated RAW264.1 cells (n=3). Statistical significance was analysed using Fisher's LSD test. Results marked in red are statistically significant in Fisher's LSD test. Multivariate Tests of Significance ($F = 7.499$, $p < 0$).

Univariate Tests of Significance for Concentration of nitrite

Effect	SS	Degr. of	MS	F	p
Intercept	62859.92	1	62859.92	26376.71	0.00
sample	1885.72	15	125.71	52.75	0.00
Error	114.39	48	2.38		

LSD test; variable Concentration of nitrite (sta-zabrze)Probabilities for Post Hoc TestsError: Between MS = 2,3832, df = 48,000

Cell No.	sample	{1}	{2}	{3}	{4}	{5}	{6}	{7}	{8}	{9}	{10}	{11}	{12}	{13}	{14}	{15}	{16}
1	K DMSO + LPS		0.000000	0.000000	0.000000	0.000008	0.000002	0.000000	0.492490	0.981278	0.000639	0.100514	0.207014	0.649640	0.862840	0.118688	0.261753
2	5B 1µM + LPS	0.000000		0.022867	0.271682	0.000000	0.000000	0.000776	0.000000	0.000000	0.000000	0.000000	0.000000	0.000000	0.000000	0.000000	0.000000
3	5B 10µM + LPS	0.000000	0.022867		0.221294	0.000000	0.000000	0.000000	0.000000	0.000000	0.000000	0.000000	0.000000	0.000000	0.000000	0.000000	0.000000
4	5B 20µM + LPS	0.000000	0.271682	0.221294		0.000000	0.000000	0.000022	0.000000	0.000000	0.000000	0.000000	0.000000	0.000000	0.000000	0.000000	0.000000
5	6B 1µM + LPS	0.000008	0.000000	0.000000	0.000000		0.690689	0.000690	0.000084	0.000009	0.188108	0.001753	0.000538	0.000002	0.000005	0.001364	0.000345
6	6B 10µM + LPS	0.000002	0.000000	0.000000	0.000000	0.690689		0.002249	0.000022	0.000002	0.089063	0.000530	0.000154	0.000000	0.000001	0.000407	0.000097
7	6B 20µM + LPS	0.000000	0.000776	0.000000	0.000022	0.000690	0.002249		0.000000	0.000000	0.000009	0.000000	0.000000	0.000000	0.000000	0.000000	0.000000
8	7B 1µM + LPS	0.492490	0.000000	0.000000	0.000000	0.000084	0.000022	0.000000		0.507296	0.004740	0.330553	0.559660	0.256340	0.391158	0.374154	0.659055
9	7B 10µM + LPS	0.981278	0.000000	0.000000	0.000000	0.000009	0.000002	0.000000	0.507296		0.000686	0.105262	0.215374	0.632900	0.844442	0.124120	0.271662
10	7B 20µM + LPS	0.000639	0.000000	0.000000	0.000000	0.188108	0.089063	0.000009	0.004740	0.000686		0.053543	0.021606	0.000153	0.000374	0.044337	0.015184
11	8B 1µM + LPS	0.100514	0.000000	0.000000	0.000000	0.001753	0.000530	0.000000	0.330553	0.105262	0.053543		0.694210	0.038174	0.070724	0.931915	0.592385
12	8B 10µM + LPS	0.207014	0.000000	0.000000	0.000000	0.000538	0.000154	0.000000	0.559660	0.215374	0.021606	0.694210		0.088940	0.152791	0.758178	0.886522
13	8B 20µM + LPS	0.649640	0.000000	0.000000	0.000000	0.000002	0.000000	0.000000	0.256340	0.632900	0.000153	0.038174	0.088940		0.778059	0.046271	0.117780
14	flawanon 1µM + LPS	0.862840	0.000000	0.000000	0.000000	0.000005	0.000001	0.000000	0.391158	0.844442	0.000374	0.070724	0.152791	0.778059		0.084365	0.196661
15	flawanon 10µM + LPS	0.118688	0.000000	0.000000	0.000000	0.001364	0.000407	0.000000	0.374154	0.124120	0.044337	0.931915	0.758178	0.046271	0.084365		0.652516
16	flawanon 20µM + LPS	0.261753	0.000000	0.000000	0.000000	0.000345	0.000097	0.000000	0.659055	0.271662	0.015184	0.592385	0.886522	0.117780	0.196661	0.652516	
